# Dynamic Multi-Swarm Differential Learning Quantum Bird Swarm Algorithm and Its Application in Random Forest Classification Model

**DOI:** 10.1155/2020/6858541

**Published:** 2020-08-07

**Authors:** Jiangnan Zhang, Kewen Xia, Ziping He, Shurui Fan

**Affiliations:** School of Electronic and Information Engineering, Hebei University of Technology, Tianjin 300401, China

## Abstract

Bird swarm algorithm is one of the swarm intelligence algorithms proposed recently. However, the original bird swarm algorithm has some drawbacks, such as easy to fall into local optimum and slow convergence speed. To overcome these short-comings, a dynamic multi-swarm differential learning quantum bird swarm algorithm which combines three hybrid strategies was established. First, establishing a dynamic multi-swarm bird swarm algorithm and the differential evolution strategy was adopted to enhance the randomness of the foraging behavior's movement, which can make the bird swarm algorithm have a stronger global exploration capability. Next, quantum behavior was introduced into the bird swarm algorithm for more efficient search solution space. Then, the improved bird swarm algorithm is used to optimize the number of decision trees and the number of predictor variables on the random forest classification model. In the experiment, the 18 benchmark functions, 30 CEC2014 functions, and the 8 UCI datasets are tested to show that the improved algorithm and model are very competitive and outperform the other algorithms and models. Finally, the effective random forest classification model was applied to actual oil logging prediction. As the experimental results show, the three strategies can significantly boost the performance of the bird swarm algorithm and the proposed learning scheme can guarantee a more stable random forest classification model with higher accuracy and efficiency compared to others.

## 1. Introduction

The concept of swarm intelligence was first proposed by Hackwood and Beni in 1992 [1]. Swarm intelligence algorithms have been proved that it can solve nondifferentiable problems, NP-hard problems, and difficult nonlinear problems which the traditional techniques cannot solve. For this reason, swarm intelligence algorithms are hotly researched in computer science and have been updated from generation to generation. For classic swarm intelligence algorithms, particle swarm optimization (PSO) [2] is used to define the basic principle and equations of the swarm intelligence algorithms. In recent years, many new swarm intelligence algorithms have been proposed, such as artificial bee colony (ABC) algorithm [3] which is inspired by the stock of food location behavior of bees. Artificial fish school algorithm (AFSA) [4] and firefly algorithm (FA) [5] are inspired by the foraging process of fish and firefly, and cat swarm optimization (CSO) [6] is developed based on vigilance and foraging behavior of cats in nature. According to the foraging behavior, vigilance behavior, and flight behavior of the bird swarms in nature, Meng et al. proposed a novel swarm intelligence algorithm called bird swarm algorithm (BSA) [7]. Meanwhile, due to these advantages above, swarm intelligence algorithms have been applied to optimize various fields, such as PSO for mutation testing problems [8], genetic algorithm (GA) for convolutional neural networks parameters [9], FA for convolutional neural network problems [10], and whale optimization algorithm (WOA) for cloud computing environments [11]. So, BSA which will be used in this paper has been widely applied to engineering optimization problems.

However, the original swarm intelligence algorithms have limitations in solving some practical problems. Hybrid strategy which is one of the main research directions to improve the performance of swarm intelligence algorithms has become a research hotspot in machine learning. Tuba and Bacanin [12] modified the exploitation process of the original seeker optimization algorithm (SOA) approach by hybridizing it with FA which overcame shortcomings and outperformed other algorithms. Strumberger et al. [13] also has proposed dynamic search tree growth algorithm (TGA) and hybridized elephant herding optimization (EHO) with ABC, and the simulation results have shown that the proposed approach was viable and effective. Yang [14] analyzed swarm intelligence algorithms by using differential evolution, dynamic systems, self-organization, and Markov chain framework. The discussions demonstrate that the hybrid algorithms have some advantages over traditional algorithms. Bacanin and Tuba [15] proposed a modified ABC based on GA, and the obtained results show that the hybrid ABC is able to provide competitive results and outperform other counterparts. Liu et al. [16] presented a multistrategy brain storm optimization (BSO) with dynamic parameter adjustment which is more competitive than other related algorithms. Peng et al. [17] has proposed FA with luciferase inhibition mechanism to improve the effectiveness of selection. The simulation results have shown that the proposed approach has the best performance in some complex functions. Peng et al. [18] also developed a hybrid approach, which is using the best neighbor-guided solution search strategy to search ABC algorithm. The experimental results indicate that the proposed ABC is very competitive and outperforms the other algorithms. It can be seen that the hybrid strategy is a strategy to successfully improve the swarm intelligence algorithm, so the BSA algorithm will be improved by hybrid strategy in this paper.

Similarly, BSA can also be applied to multiple fields, especially in the field of parameter estimation, and hybrid strategy is also the improvement method for BSA. In 2017, Xu et al. [19] proposed improved boundary BSA (IBBSA) for chaotic system optimization of the Lorenz system and the coupling motor system. However, the improved boundary learning strategy has randomness, which makes IBBSA generalization performance not high. Yang and Liu [20] introduced the dynamic weight into the foraging formula of BSA (IBSA) which provides a solution for problem that anti-same-frequency interference of shipborne radar. The results have shown that the dynamic weight is just introduced into the foraging formula of BSA, but IBSA ignored the impact of population initialization. Wang et al. [21] designed a strategy named “disturbing the local optimum” for helping the original BSA converge to the global optimal solution faster and more stably. However, “disturbing the local optimum” also has randomness, which makes the generalization performance of improved BSA not very well.

Like many swarm intelligence algorithms, BSA is also faced with the problem of being trapped in local optima and slow convergence. These disadvantages limit the wider application of BSA. In this paper, a dynamic multi-swarm differential learning quantum BSA called DMSDL-QBSA is proposed, which introduced three hybrid strategies into the original BSA to improve its effectiveness. Motivated by the defect of insufficient generalization ability in the literature [19, 21], we will first establish a dynamic multi-swarm bird swarm algorithm (DMS-BSA) and merge the differential evolution operator into each sub-swarm of the DMS-BSA, and it improves the local search capability and global search capability of foraging behavior. Second, according to the contempt for the impact of population initialization in the literature [20], they used quantum behavior to optimize the particle swarm optimization in order to obtain a good ability to jump out of global optimum; we will use the quantum system to initialize the search space of the bird. Consequently, it improves the convergence rate of the whole population and avoids BSA into a local optimum. In order to validate effectiveness of the proposed method, we have evaluated the performance of DMSDL-QBSA on classical benchmark functions and CEC2014 functions including unimodal and multimodal functions in comparison with the state-of-the-art methods and new popular algorithms. The experimental results have shown that the three improvement strategies are able to significantly boost the performance of BSA.

Based on the DMSDL-QBSA, an effective hybrid random forest (RF) model for actual oil logging prediction is established, called DMSDL-QBSA-RF approach. RF has the characteristics of being nonlinear and anti-interference [22]. In addition, it can decrease the possibility of overfitting which often occurs in actual logging. RF has been widely used in various classification problems, but it has not yet been applied to the field of actual logging. Parameter estimation is a prerequisite to accomplish the RF classification model. The two key parameters of RF are the number of decision trees and the number of predictor variables; the former is called [−100,100]^*D*^, and the latter is called *m*_try_. Meanwhile, parameter estimation of the model is a complex optimization problem that traditional methods might fail to solve. Many works have proposed to use swarm intelligence algorithms to find the best parameters of the RF model. Ma and Fan [23] adopted AFSA and PSO to optimize the parameters of the RF. Hou et al. [24] used the DE to obtain an optimal set of initial parameters for RF. Liu et al. [25] compared genetic algorithms, simulated annealing, and hill climbing algorithms to optimize the parameters of the RF. From these papers, we can see that metaheuristic algorithm must be suitable for this problem. In this study, the DMSDL-QBSA was used to optimize the two key parameters that can improve the accuracy without overfitting for RF. When investigating the performance of the DMSDL-QBSA-RF classification model compared with 3 swarm intelligence algorithm-based RF methods, 8 two-dimensional UCI datasets are applied. As the experimental results show, the proposed learning scheme can guarantee a more stable RF classification model with higher predictive accuracy compared to other counterparts. The rest of the paper is organized as follows:

- In order to achieve a better balance between efficiency and velocity for BSA, we have studied the effects of four different hybrid strategies of the dynamic multi-swarm method, differential evolution, and quantum behavior on the performance of BSA.
- The proposed DMSDL-QBSA has successfully optimized *n*_tree_ and *m*_try_ setting problem of RF. The resulting hybrid classification model has been rigorously evaluated on oil logging prediction.
- The proposed hybrid classification model delivers better classification performance and offers more accurate and faster results when compared to other swarm intelligence algorithm-based RF models.

## 2. Bird Swarm Algorithm and Its Improvement

### 2.1. Bird Swarm Algorithm Principle

BSA, as proposed by Meng et al. in 2015, is a new intelligent bionic algorithm based on multigroup and multisearch methods; it mimics the birds' foraging behavior, vigilance behavior, and flight behavior, and employs this swarm intelligence to solve the optimization problem. The bird swarm algorithm can be simplified by the five rules:

  Rule 1: each bird can switch between vigilant behavior and foraging behavior, and both bird forages and keeps vigilance is mimicked as random decisions.
  Rule 2: when foraging, each bird records and updates its previous best experience and the swarms' previous best experience with food patches. The experience can also be used to search for food. Instant sharing of social information is across the group.
  Rule 3: when keeping vigilance, each bird tries to move towards the center of the swarm. This behavior may be influenced by disturbances caused by swarm competition. Birds with more stocks are more likely to be near swarm's centers than birds with lease stocks.
  Rule 4: birds fly to another place regularly. When flying to another location, birds often switch between production and shrubs. The bird with the most stocks is the producer, and the bird with the least is a scrounger. Other birds with the highest and lowest reserves are randomly selected for producers and scroungers.
  Rule 5: producers actively seek food. Scroungers randomly follow producers looking for food.

According to Rule 1, we define that the time interval of each bird flight behavior FQ, the probability of foraging behavior *P*(*P* ∈ (0,1)), and a uniform random number *δ* ∈ (0,1).

1. Foraging behavior
2. If the number of iteration is less than FQ and *δ* ≤ *P*, the bird will be the foraging behavior. Rule 2 can be written mathematically as follows:

(1) $$\begin{array}{c} x_{i,j}^{t+1}=x_{i,j}^{t}+\left(p_{i,j}^{t}-x_{i,j}^{t}\right)\times C\times \text{rand}\left(0,1\right)+\left(g_{j}^{t}-x_{i,j}^{t}\right)\times S\times \text{rand}\left(0,1\right), \end{array}$$

  where *C* and *S* are two positive numbers; the former is called cognitive accelerated coefficients, and the latter is called social accelerated coefficients. Here, *p*_*i*,*j*_ is the *i*-th bird's best previous position and *g*_*j*_ is the best previous swarm's position.
(2) Vigilance behavior
  If the number of iteration is less than FQ and *δ* > *P*, the bird will be the vigilance behavior. Rule 3 can be written mathematically as follows:

(2) $$\begin{array}{c} x_{i,j}^{t+1}=x_{i,j}^{t}+A_{1}\left({\text{mean}}_{j}^{t}-x_{i,j}^{t}\right)\times \text{rand}\left(0,1\right)+A_{2}\left(p_{k,j}^{t}-x_{i,j}^{t}\right)\times \text{rand}\left(-1,1\right), \end{array}$$

(3) $$\begin{array}{c} A_{1}=a_{1}\times \;\exp\left(-\frac{p{\text{Fit}}_{i}}{\text{sumFit}+\varepsilon }\times N\right), \end{array}$$

(4) $$\begin{array}{c} A_{2}=a_{2}\times \;\exp\left(\left(\frac{p{\text{Fit}}_{i}-p{\text{Fit}}_{k}}{\left|p{\text{Fit}}_{k}-p{\text{Fit}}_{i}\right|+\varepsilon }\right)\times \frac{N\times p{\text{Fit}}_{k}}{\text{sumFit}+\varepsilon }\right), \end{array}$$

  where *a*_1_ and *a*_2_ are two positive constants in [0,2], *p*Fit_*i*_ is the best fitness value of *i*-th bird, and sumFit is the sum of the swarms' best fitness value. Here, *ε*, which is used to avoid zero-division error, is the smallest constant in the computer. mean_*j*_ denotes the *j*-th element of the whole swarm's average position.
(3) Flight behavior
  If the number of iteration equals FQ, the bird will be the flight behavior which can be divided into the behaviors of the producers and scroungers by fitness. Rule 3 and Rule 4 can be written mathematically as follows:

(5) $$\begin{array}{c} x_{i,j}^{t+1}=x_{i,j}^{t}+\text{randn}\left(0,1\right)\times x_{i,j}^{t}, \end{array}$$

(6) $$\begin{array}{c} x_{i,j}^{t+1}=x_{i,j}^{t}+\left(x_{k,j}^{t}-x_{i,j}^{t}\right)\times \text{FL}\times \text{rand}\left(0,1\right), \end{array}$$

where FL (FL ∈ [0,2]) means that the scrounger would follow the producer to search for food.

### 2.2. The Bird Swarm Algorithm Based on Dynamic Multi-Swarm Method, Differential Evolution, and Quantum Behavior

#### 2.2.1. The Foraging Behavior Based on Dynamic Multi-Swarm Method

Dynamic multi-swarm method has been widely used in real-world applications, because it is efficient and easy to implement. In addition, it is very common in the improvement of swarm intelligent optimization, such as coevolutionary algorithm [26], the framework of evolutionary algorithms [27], multiobjective particle swarm optimization [28], hybrid dynamic robust [29], and PSO algorithm [30, 31]. However, the PSO algorithm is easy to fall into the local optimum and its generalization performance is not high. Consequently, motivated by these literature studies, we will establish a dynamic multi-swarm bird swarm algorithm (DMS-BSA), and it improves the local search capability of foraging behavior.

In DMS-PSO, the whole population is divided into many small swarms, which are often regrouped by using various reorganization plans to exchange information. The velocity update strategy is

(7) $$\begin{array}{c} v_{i,j}^{t+1}=\omega v_{i,j}^{t}+c_{2}r_{2}\left(l_{i,j}^{t}-x_{i,j}^{t}\right), \end{array}$$

where *l*_*i*,*j*_ is the best historical position achieved within the local community of the *i*-th particle.

According to the characteristic of equation (1), we can see that the foraging behavior formula of BSA is similar to the particle velocity update formula of PSO. So, according to the characteristic of equation (7), we can get the improved foraging behavior formula as follows:

(8) $$\begin{array}{c} x_{i,j}^{t+1}=x_{i,j}^{t}+\left(GV_{i,j}^{t}-x_{i,j}^{t}\right)\times C\times \text{rand}\left(0,1\right), \end{array}$$

where GV is called the guiding vector.

The dynamic multi-swarm method is used to improve the local search capability, while the guiding vector GV can enhance the global search capability of foraging behavior. Obviously, we need to build a good guiding vector.

#### 2.2.2. The Guiding Vector Based on Differential Evolution

Differential evolution (DE) is a powerful evolutionary algorithm with three differential evolution operators for solving the tough global optimization problems [32]. Besides, DE has got more and more attention of scholars to evolve and improve in evolutionary computation, such as hybrid multiple crossover operations [33] and proposed DE/neighbor/1 [34], due to its excellent global search capability. From these literature studies, we can see that DE has a good global search capability, so we will establish the guiding vector GV based on differential evolution operator to improve the global search capability of foraging behavior. The detailed implementation of GV is presented as follows:

1. Differential mutation
2. According to the characteristic of equation (8), the “DE/best/1”, “DE/best/2,” and “DE/current-to-best/1” mutation strategies are suitable. In the experiments of the literature [31], they showed that the “DE/best/1” mutation strategy is the most suitable in DMS-PSO, so we choose this mutation strategy in BSA. And the “DE/lbest/1” mutation strategy can be written as follows:
3. DE/lbest/1：

(9) $$\begin{array}{c} v_{i,j}^{t}=l_{i,j}^{t}+F\times \left(p_{r1,j}^{t}-p_{r2,j}^{t}\right). \end{array}$$

  Note that some components of the mutant vector *v*_*i*,*j*_ may violate predefined boundary constraints. In this case, the boundary processing is used. It can be expressed as follows:

(10) $$\begin{array}{c} v_{i,j}^{t}=\left\{\begin{array}{cc} x_{\text{lb}}, & v_{i,j}^{t}<x_{\text{lb}},\\ x_{\text{ub}}, & v_{i,j}^{t}\geq x_{\text{ub}}. \end{array}\right. \end{array}$$

(2) Crossover
  After differential mutation, a binomial crossover operation exchanges some components of the mutant vector *v*_*i*,*j*_ with the best previous position *p*_*i*,*j*_ to generate the target vector *u*_*i*,*j*_. The process can be expressed as

(11) $$\begin{array}{c} u_{i,j}^{t}=\left\{\begin{array}{cc} v_{i,j}^{t}, & \text{rand}\leq \text{CR or }j=\text{rand}\left(i\right),\\ p_{i,j}^{t}, & \text{otherwise.} \end{array}\right. \end{array}$$

(3) Selection
  Because the purpose of BSA is to find the best fitness, a selection operation chooses a vector accompanied a better fitness to enter the next generation to generate the selection operator, namely, guiding vector GV. The process can be expressed as follows:
  Choose a vector with better fitness to enter the next generation

(12) $$\begin{array}{c} {\text{GV}}_{i,j}^{t+1}=\left\{\begin{array}{cc} u_{i,j}^{t}, & \text{fitness}\left(u_{i,j}^{t}\right)\leq \text{fitness}\left(x_{i,j}^{t}\right),\\ p_{i,j}^{t}, & \text{otherwise.} \end{array}\right. \end{array}$$

#### 2.2.3. The Initialization of Search Space Based on Quantum Behavior

Quantum behavior is a nonlinear and excellent superposition system. With its simple and effective characteristics and good performance in global optimization, it has been applied to optimize many algorithms, such as particle swarm optimization [35] and pigeon-inspired optimization algorithm [36]. Consequently, according to the literature studies and its excellent global optimization performance, we use the quantum system to initialize the search space of the bird.

Quantum-behaved particle position can be written mathematically as follows:

(13) $$\begin{array}{c} x^{t+1}=p^{t}\pm \frac{L^{t}}{2}\ln\frac{1}{u}, \end{array}$$

(14) $$\begin{array}{c} p^{t}=c_{1}\times p{\text{best}}^{t}+\left(1-c_{1}\right)\times g{\text{best}}^{t}, \end{array}$$

(15) $$\begin{array}{c} L^{t}=2\beta \left|P^{t}-x^{t}\right|. \end{array}$$

According to the characteristics of equations (13)–(15), we can get the improved search space initialization formula as follows:

(16) $$\begin{array}{c} x_{i}^{t}=\text{lb}+\left(\text{ub}-\text{lb}\right)\times \text{rand,} \end{array}$$

(17) $$\begin{array}{c} x_{i}^{t+1}=x_{i}^{t}\pm \left|m\text{best}-x_{i}^{t}\right|\times \;\ln\frac{1}{u}, \end{array}$$

where *β* is a positive number, which can be, respectively, called as a contraction expansion factor. Here, *x*_*i*_^*t*^ is the position of the particle at the previous moment and mbest is the average value of the best previous positions of all the birds (Algorithm 1).

#### 2.2.4. Procedures of the DMSDL-QBSA

In Sections 2.2.1–2.2.3, in order to improve the local search capability and the global search capability on BSA, this paper has improved the BSA in three parts:

1. In order to improve the local search capability of foraging behavior on BSA, we put forward equation (8) based on the dynamic multi-swarm method.
2. In order to get the guiding vector to improve the global search capability of foraging behavior on BSA, we put forward equations (9), (11), and (12) based on differential evolution.
3. In order to expand the initialization search space of the bird to improve the global search capability on BSA, we put forward equations (16) and (17) based on quantum behavior.

Finally, the steps of DMSDL-QBSA can be shown in Algorithm 1.

### 2.3. Simulation Experiment and Analysis

This section presents the evaluation of DMSDL-QBSA using a series of experiments on benchmark functions and CEC2014 test functions. All experiments in this paper are implemented using the following: MATLAB R2014b; Win 7 (64-bit); Inter (R) Core (TM) i5-2450M; CPU @2.50 GHz; 4.00 GB RAM. To obtain fair results, all the experiments were conducted under the same conditions. The number of the population size is set as 30 in these algorithms. And each algorithm runs 30 times independently for each function.

#### 2.3.1. Benchmark Functions and CEC 2014 Test Functions

When investigating the effective and universal performance of DMSDL-QBSA compared with several hybrid algorithms and popular algorithms, 18 benchmark functions and CEC2014 test functions are applied. In order to test the effectiveness of the proposed DMSDL-QBSA, 18 benchmark functions [37] are adopted, and all of which have an optimal value of 0. The benchmark functions and their searching ranges are shown in Table 1. In this test suite, *f*_1_ − *f*_9_ are unimodal functions. These unimodal functions are usually used to test and investigate whether the proposed algorithm has a good convergence performance. Then, *f*_10_ − *f*_18_ are multimodal functions. These multimodal functions are used to test the global search capability of the proposed algorithm. The smaller the fitness value of functions, the better the algorithm performs. Furthermore, in order to better verify the comprehensive performance of DMSDL-QBSA in a more comprehensively manner, another 30 complex CEC2014 benchmarks are used. The CEC2014 benchmark functions are simply described in Table 2.

#### 2.3.2. Parameter Settings

In order to verify the effectiveness and generalization of the proposed DMSDL-QBSA, the improved DMSDL-QBSA is compared with several hybrid algorithms. These algorithms are BSA [7], DE [32], DMSDL-PSO [31], and DMSDL-BSA. Another 5 popular intelligence algorithms, such as grey wolf optimizer (GWO) [38], whale optimization algorithm (WOA) [39], sine cosine algorithm (SCA) [40], grasshopper optimization algorithm (GOA) [41], and sparrow search algorithm (SSA) [42], are used to compare with DMSDL-QBSA. These algorithms represented state-of-the-art can be used to better verify the performance of DMSDL-QBSA in a more comprehensively manner. For fair comparison, the number of populations of all algorithms is set to 30, respectively, and other parameters of all algorithms are set according to their original papers. The parameter settings of these involved algorithms are shown in Table 3 in detail.

#### 2.3.3. Comparison on Benchmark Functions with Hybrid Algorithms

According to Section 2.2, three hybrid strategies (dynamic multi-swarm method, DE, and quantum behavior) have been combined with the basic BSA method. When investigating the effectiveness of DMSDL-QBSA compared with several hybrid algorithms, such as BSA, DE, DMSDL-PSO, and DMSDL-BSA, 18 benchmark functions are applied. Compared with DMSDL-QBSA, quantum behavior dynamic is not used in the dynamic multi-swarm differential learning bird swarm algorithm (DMSDL-BSA). The number of function evaluations (FEs) is 10000. We selected two different dimension's sizes (Dim). Dim = 10 is the typical dimensions for the benchmark functions. And Dim = 2 is for RF has two parameters that need to be optimized, which means that the optimization function is 2-dimensional.

**Figure 1:**
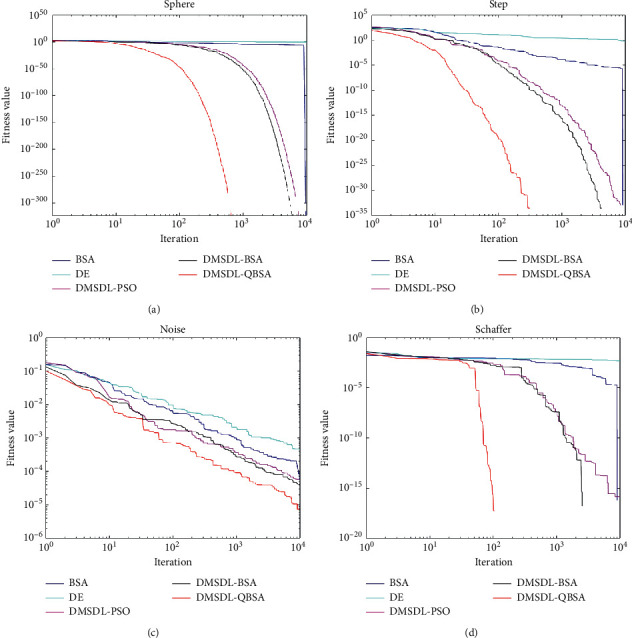
Fitness value curves of 5 hybrid algorithms on (a)*f*_1_; (b)*f*_5_; (c)*f*_6_; and (d)*f*_9_ (Dim = 2).

**Figure 2:**
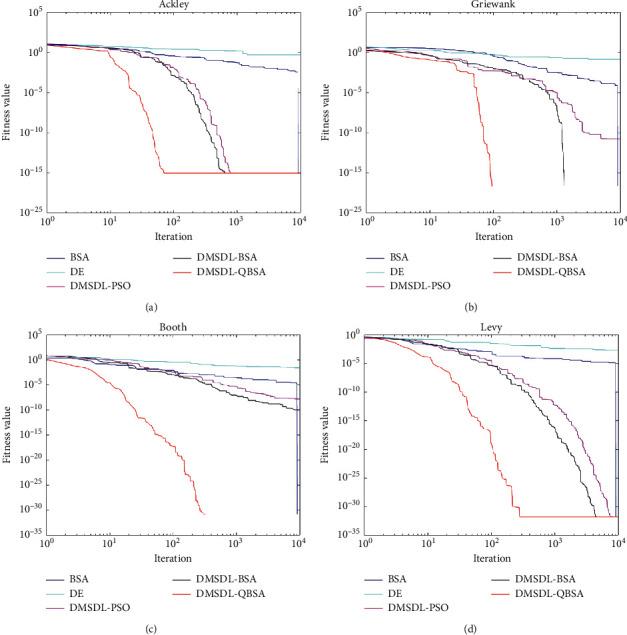
Fitness value curves of 5 hybrid algorithms on (a)*f*_12_; (b)*f*_13_; (c)*f*_17_; and (d)*f*_18_ (Dim = 2).

The fitness value curves of a run of several algorithms on about eight different functions are shown in Figures 1 and 2, where the horizontal axis represents the number of iterations and the vertical axis represents the fitness value. We can obviously see the convergence speeds of several different algorithms. The maximum value (Max), the minimum value (Min), the mean value (Mean), and the variance (Var) obtained by several benchmark algorithms are shown in Tables 4–7, where the best results are marked in bold.  Table 4 and 5 show the performance of the several algorithms on unimodal functions when Dim = 10 and 2, and Table 6 and 7 show the performance of the several algorithms on multimodal functions when Dim = 10 and 2.

  (1) Unimodal functions
  From the numerical testing results on 8 unimodal functions in Table 4, we can see that DMSDL-QBSA can find the optimal solution for all unimodal functions and get the minimum value of 0 on *f*_1_, *f*_2_, *f*_3_, *f*_7_, and *f*_8_. Both DMSDL-QBSA and DMSDL-BSA can find the minimum value. However, DMSDL-QBSA has the best mean value and variance on each function. The main reason is that DMSDL-QBSA has better population diversity during the initialization period. In summary, the DMSDL-QBSA has best performance on unimodal functions compared to the other algorithms when Dim = 10. Obviously, the DMSDL-QBSA has a relatively well convergence speed.
  The evolution curves of these algorithms on four unimodal functions *f*_1_, *f*_5_, *f*_6_, and *f*_9_ are drawn in Figure 1. It can be detected from the figure that the curve of DMSDL-QBSA descends fastest in the number of iterations that are far less than 10000 times. For *f*_1_, *f*_5_, and *f*_9_ case, DMSDL-QBSA has the fastest convergence speed compared with other algorithms. However, the original BSA got the worst solution because it is trapped in the local optimum prematurely. For function *f*_6_, these algorithms did not find the value 0. However, the convergence speed of the DMSDL-QBSA is significantly faster than other algorithms in the early stage and the solution eventually found is the best. Overall, owing to enhance the diversity of population, DMSDL-QBSA has a relatively excellent convergence speed when Dim = 2.
  According to the results of Table 5, DMSDL-QBSA gets the best performance on these 8 unimodal functions when Dim = 2. DMSDL-QBSA finds the minimum value of 0 on *f*_1_, *f*_2_, *f*_3_, *f*_4_, *f*_5_, *f*_7_, *f*_8_, and *f*_9_. DMSDL-QBSA has better performance on *f*_2_, *f*_4_, and *f*_9_ compared with DMSDL-BSA and DMSDL-PSO. DE does not perform well on these functions, but BSA performs relatively well on *f*_1_, *f*_3_, *f*_6_, *f*_7_, *f*_8_, and *f*_9_. The main reason is that BSA has a better convergence performance in the early search. Obviously, DMSDL-QBSA can find the best two parameters for RF that need to be optimized.
(2) Multimodal functions
  From the numerical testing results on 8 multimodal functions in Table 6, we can see that DMSDL-QBSA can find the optimal solution for all multimodal functions and get the minimum value of 0 on *f*_11_, *f*_13_, and *f*_16_. DMSDL-QBSA has the best performance on *f*_11_, *f*_12_, *f*_13_, *f*_14_, *f*_15_, and *f*_16_. BSA works the best on *f*_10_. DMSDL-PSO performs not very well. And DMSDL-QBSA has the best mean value and variance on most functions. The main reason is that DMSDL-QBSA has a stronger global exploration capability based on the dynamic multi-swarm method and differential evolution. In summary, the DMSDL-QBSA has relatively well performance on unimodal functions compared to the other algorithms when typical Dim = 10. Obviously, the DMSDL-QBSA has relatively well global search capability.

The evolution curves of these algorithms on four multimodal functions *f*_12_, *f*_13_, *f*_17_, and *f*_18_ when Dim = 2 are depicted in Figure 2. We can see that DMSDL-QBSA can find the optimal solution in the same iteration. For *f*_13_ and *f*_17_ case, DMSDL-QBSA continues to decline. However, the original BSA and DE get parallel straight lines because of their poor global convergence ability. For functions *f*_12_ and *f*_18_, although DMSDL-QBSA also trapped the local optimum, it find the minimum value compared to other algorithms. Obviously, the convergence speed of the DMSDL-QBSA is significantly faster than other algorithms in the early stage, and the solution eventually found is the best. In general, owing to enhance the diversity of population, DMSDL-QBSA has a relatively balanced global search capability when Dim = 2.

Furthermore, from the numerical testing results on nine multimodal functions in Table 7, we can see that DMSDL-QBSA has the best performance on *f*_11_, *f*_12_, *f*_13_, *f*_14_, *f*_16_, *f*_17_, and *f*_18_. DMSDL-QBSA gets the minimum value of 0 on *f*_11_, *f*_13_, *f*_16_, and *f*_17_. BSA has got the minimum value of 0 on *f*_11_, *f*_13_, and *f*_17_. DE also has not got the minimum value of 0 on any functions. DMSDL-BSA has got the minimum value of 0 on *f*_11_ and *f*_13_. In summary, the DMSDL-QBSA has a superior global search capability on most multimodal functions when Dim = 2. Obviously, DMSDL-QBSA can find the best two parameters for RF that need to be optimized, because of its best global search capability.

In this section, it can be seen from Figures 1 and 2 and Tables 4–7 that DMSDL-QBSA can obtain the best function values for most cases. It indicates that the hybrid strategies of BSA, dynamic multi-swarm method, DE, and quantum behavior operators, lead to the bird moves towards the best solutions. And DMSDL-QBSA has well ability of searching for the best two parameters for RF with higher accuracy and efficiency.

#### 2.3.4. Comparison on Benchmark Functions with Popular Algorithms

When comparing the timeliness and applicability of DMSDL-QBSA compared with several popular algorithms, such as GWO, WOA, SCA, GOA, and SSA, 18 benchmark functions are applied. And GWO, WOA, GOA and SSA are swarm intelligence algorithms. In this experiment, the dimension's size of these functions is10. The number of function evaluations (FEs) is100000. The maximum value (Max), the minimum value (Min), the mean value (Mean), and the variance (Var) obtained by several different algorithms are shown in Tables 8 and 9, where the best results are marked in bold.

From the test results in Table 8, we can see that DMSDL-QBSA has the best performance on each unimodal function. GWO finds the value 0 on *f*_1_, *f*_2_, *f*_3_, *f*_7_, and *f*_8_. WOA obtains 0 on *f*_1_, *f*_2_, and *f*_7_. SSA works the best on *f*_1_ and *f*_7_. With the experiment of multimodal function evaluations, Table 9 shows that DMSDL-QBSA has the best performance on *f*_11_, *f*_12_, *f*_13_, *f*_14_, *f*_15_, *f*_16_, and *f*_18_. SSA has the best performance on *f*_10_. GWO gets the minimum on *f*_11_. WOA and SCA obtains the optimal value on *f*_11_ and *f*_13_. Obviously, compared with these popular algorithms, DMSDL-QBSA is a competitive algorithm for solving several functions and the swarm intelligence algorithms perform better than other algorithms. The results of Tables 8 and 9 show that DMSDL-QBSA has the best performance on the most test benchmark functions.

#### 2.3.5. Comparison on CEC2014 Test Functions with Hybrid Algorithms

When comparing the comprehensive performance of proposed DMSDL-QBSA compared with several hybrid algorithms, such as BSA, DE, DMSDL-PSO, and DMSDL-BSA, 30 CEC2014 test functions are applied. In this experiment, the dimension's size (Dim) is set to 10. The number of function evaluations (FEs) is 100000. Experimental comparisons included the maximum value (Max), the minimum value (Min), the mean value (Mean), and the variance (Var) are given in Tables 10 and 11, where the best results are marked in bold.

Based on the mean value (Mean), on the CEC2014 test functions, DMSDL-QBSA has the best performance on *F*_2_, *F*_3_, *F*_4_, *F*_6_, *F*_7_, *F*_8_, *F*_9_, *F*_10_, *F*_11_, *F*_15_, *F*_16_, *F*_17_, *F*_21_, *F*_26_, *F*_27_, *F*_29_, and *F*_30_. DMSDL-BSA does show an advantage on *F*_1_, *F*_12_, *F*_13_, *F*_14_, *F*_18_, *F*_19_, *F*_20_, *F*_24_, *F*_25_, and *F*_28_. According to the results, we can observe that DMSDL-QBSA can *f*ind the minimal value on 17 CEC2014 test *f*unctions. DMSDL-BSA gets the minimum value on *F*_1_, *F*_12_, *F*_13_, *F*_14_, *F*_18_, *F*_19_, *F*_24_, and *F*_30_, and DMSDL-PSO obtains the minimum value on *F*_4_, *F*_7_, and *F*_23_. Owing to enhance the capability o*f* exploitation, DMSDL-QBSA is better than DMSDL-BSA and DMSDL-PSO on most functions. From the results of tests, it can be seen that DMSDL-QBSA performs better than BSA, DE, DMSDL-PSO, and DMSDL-BSA. It can be observed that DMSDL-QBSA obtains optimal value. It can be concluded that DMSDL-QBSA has better global search ability and better robustness on these test suites.

## 3. Optimize RF Classification Model Based on Improved BSA Algorithm

### 3.1. RF Classification Model

RF, as proposed by Breiman et al., is an ensemble learning model based on bagging and random subspace methods. The whole modeling process includes building decision trees and decision processes. The process of constructing decision trees is mainly composed of *n*_tree_ decision trees, and each of which consists of nonleaf nodes and leaf nodes. The leaf node is a child node of the node branch. It is supposed that the dataset has *M* attributes. When each leaf node of the decision tree needs to be segmented, the *m*_try_ attributes are randomly selected from the *M* attributes as the reselected splitting variables of this node. This process can be defined as follows:

(18) $$\begin{array}{c} S_{j}=\left\{P_{i}\left|i=1,2,...,m_{\text{try}}\right.\right\}, \end{array}$$

where *S*_*j*_ is the splitting variable of the *j*-th leaf node of the decision tree, and *P*_*i*_ is the probability that *m*_try_ reselected attributes are selected as the splitting attribute of the node.

The nonleaf node is a parent node that classifies training data as a left or right child node. The function of *k*-th decision tree is as follows:

(19) $$\begin{array}{c} h_{k}\left(c\left|x\right.\right)=\left\{\begin{array}{cc} 0, & f_{l}\left(x_{k}\right)<f_{l}\left(x_{k}\right)+\tau ,\\ 1, & f_{l}\left(x_{k}\right)\geq f_{l}\left(x_{k}\right)+\tau , \end{array}\right.\;l=1,2,\ldots ,n_{\text{tree}}, \end{array}$$

where *c*={0 or 1}, where the symbol 0 indicates that the *k*-th row of data is classified as a negative label and the symbol 1 indicates that the *k*-th row of data is classified as a positive label. Here, *f*_*l*_ is the training function of the *l*-th decision tree based on the splitting variable *S*. *X*_*k*_ is the *k*-th row of data in the dataset by random sampling with replacement. The symbol *τ* is a positive constant, which is used as the threshold value of the training decision.

When decision processes are trained, each row of data will be input into a leaf node of each decision tree. The average of *n*_tree_ decision tree classification results is used as the final classification result. This process can be written mathematically as follows:

(20) $$\begin{array}{c} c_{k}=l\times \frac{1}{n_{\text{tree}}}\sum_{k=1}^{n_{\text{tree}}}h_{k}\left(c\left|x\right.\right), \end{array}$$

where *l* is the number of decision trees which judged *k*-th row of data as *c*.

**Figure 3:**
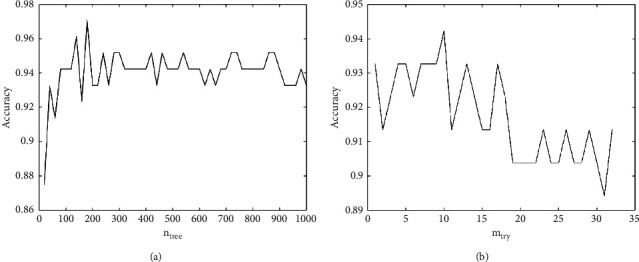
The effect of the two parameters on the performance of RF models: (a) the effect of *n*_tree_; (b) the effect of *m*_try_.

From the above principle, we can see that it is mainly necessary to determine two parameters of *n*_tree_ and *m*_try_ in the RF modeling process. In order to verify the influence of these two parameters on the classification accuracy of the RF classification model, the Ionosphere dataset is used to test the influence of the two parameters on the performance of the RF model, as shown in Figure 3, where the horizontal axis represents *n*_tree_ and *m*_try_, respectively, and the vertical axis represents the accuracy of the RF classification model.

1. Parameter analysis of *n*_tree_
2. When the number of predictor variables *m*_try_ is set to 6, the number of decision trees *n*_tree_ is cyclically set from 0 to 1000 at intervals of 20. And the evolutionary progress of RF classification model accuracy with the change of *n*_tree_ is shown in Figure 3(a). From the curve in Figure 3(a), we can see that the accuracy of RF is gradually improved with the increase of the number *N* of decision trees. However, when the number of decision trees *n*_tree_ is greater than a certain value, the improvement of RF performance has become gentle without obvious improvement, but the running time becomes longer.
3. Parameter analysis of *m*_try_
4. When the number of decision trees *n*_tree_ is set to 500, the number of predictor variables *m*_try_ is cyclically set from 1 to 32. The limit of *m*_try_ is set to 32, because the number of attributes of the Ionosphere dataset is 32. And the obtained curve of RF classification model accuracy with *m*_try_ transformation is shown in Figure 3(b). And we can see that with the increase of the splitting property of the selection, the classification performance of RF is gradually improved, but when the number of predictor variables *m*_try_ is greater than 9, the RF generates overfitting and the accuracy of RF begins to decrease. The main reason is that too many split attributes are selected, which resulted in the same splitting attributes which are owned by a large number of decision trees. This reduced the diversity of decision trees.

In summary, for the RF classification model to obtain the ideal optimal solution, the selection of the number of decision trees *n*_tree_ and the number of predictor variables *m*_try_ are very important. And the classification accuracy of the RF classification model can only be optimized by the comprehensive optimization of these two parameters. So, it is necessary to use the proposed algorithm to find a suitable set of RF parameters. Next, we will optimize the RF classification model by the improved BSA proposed in Section 2.

### 3.2. RF Model Based on an Improved Bird Swarm Algorithm

Improved bird swarm algorithm optimized RF classification model (DMSDL-QBSA-RF) is based on the improved bird swarm algorithm optimized the RF classification model and introduced the training dataset into the training process of the RF classification model, finally getting the DMSDL-QBSA-RF classification model. The main idea is to construct a two-dimensional fitness function containing RF's two parameters *n*_tree_ and *m*_try_ as the optimization target of DMSDL-QBSA, so as to obtain a set of grouping parameters and make the RF classification model obtain the best classification accuracy. The specific algorithm steps are shown as in Algorithm 2.

### 3.3. Simulation Experiment and Analysis

In order to test the performance of the improved DMSDL-QBSA-RF classification model, we compare the improved classification model with the standard RF model, BSA-RF model, and DMSDL-BSA-RF model on 8 two-dimensional UCI datasets. The DMSDL-BSA-RF classification model is an RF classification model optimized by BSA without quantum behavior. In our experiment, each of datasets is divided into two parts: 70% of the dataset is as training set and the remaining 30% is as a test set. The average classification accuracies of 10 independent runs of each model are recorded in Table 12, where the best results are marked in bold.

From the accuracy results in Table 12, we can see that the DMSDL-QBSA-RF classification model can get best accuracy on each UCI dataset except magic dataset. And the DMSDL-BSA-RF classification model has got best accuracy on magic dataset. Then, compared with the standard RF model, the accuracy of the DMSDL-QBSA-RF classification model can get better accuracy which is increased by about 10%. Finally, the DMSDL-QBSA-RF classification model has got the best accuracy on appendicitis dataset which is up to 93.55%. In summary, the DMSDL-QBSA-RF classification model has validity on most datasets and a good performance on them.

## 4. Oil Layer Classification Application

### 4.1. Design of Oil Layer Classification System

**Figure 4:**
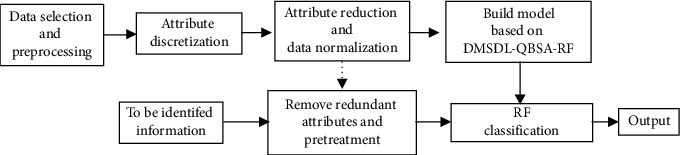
Block diagram of the oil layer classification system based on DMSDL-QBSA-RF.

The block diagram of the oil layer classification system based on the improved DMSDL-QBSA-RF is shown in Figure 4. The oil layer classification can be simplified by the following five steps:

 
*Step 1*. The selection of the actual logging datasets is intact and full-scale. At the same time, the datasets should be closely related to rock sample analysis. The dataset should be relatively independent. The dataset is randomly divided into two parts of training and testing samples.
 
*Step 2*. In order to better understand the relationship between independent variables and dependent variables and reduce the sample information attribute, the dataset continuous attribute should be discretized by using a greedy algorithm.
 
*Step 3*. In order to improve the calculation speed and classification accuracy, we use the covering rough set method [43] to realize the attribute reduction. After attribute reduction, normalization of the actual logging datasets is carried out to avoid computational saturation.
 
*Step 4*. In the DMSDL-QBSA-RF layer classification model, we input the actual logging dataset after attribute reduction, use a DMSDL-QBSA-RF layer classification algorithm to train, and finally get the DMSDL-QBSA-RF layer classification model.
 
*Step 5*. The whole oil section is identified by the trained DMSDL-QBSA-RF layer classification model, and we output the classification results.

In order to verify the application effect of the DMSDL-QBSA-RF layer classification model, we select three actual logging datasets of oil and gas wells to train and test.

### 4.2. Practical Application

In Section 2.3, the performance of the proposed DMSDL-QBSA is simulated and analyzed on benchmark functions. And in Section 3.3, the effectiveness of the improved RF classification model optimized by the proposed DMSDL-QBSA is tested and verified on two-dimensional UCI datasets. In order to test the application effect of the improved DMSDL-QBSA-RF layer classification model, three actual logging datasets are adopted and recorded as mathematical problems in engineering *W*1, *W*2, and *W*3. The *W*1 is a gas well in Xian (China), the W2 is a gas well in Shanxi (China), and the *W*3 is an oil well in Xinjiang (China). The depth and the corresponding rock sample analysis samples of the three wells selected in the experiment are as shown in Table 13.

**Figure 5:**
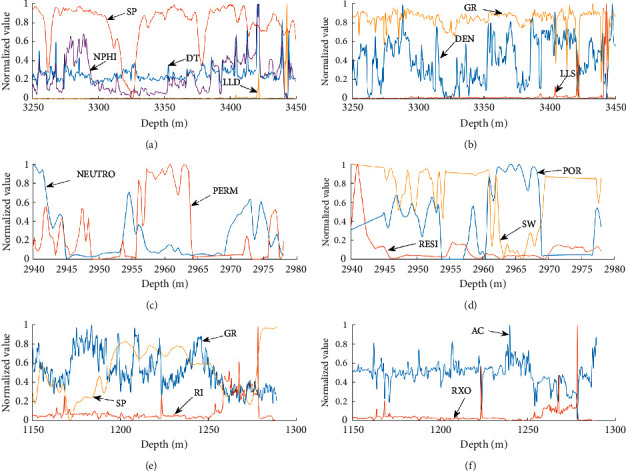
The normalized curves of attributes: (a) and (b) attribute normalization of *W*1; (c) and (d) attribute normalization of *W*2; (e) and (f) attribute normalization of *W*3.

Attribute reduction on the actual logging datasets is performed before the training of the DMSDL-QBSA-RF classification model on the training dataset, as shown in Table 14. Then, these attributes are normalized as shown in Figure 5, where the horizontal axis represents the depth and the vertical axis represents the normalized value.

The logging dataset after attribute reduction and normalization is used to train the oil and gas layer classification model. In order to measure the performance of the DMSDL-QBSA-RF classification model, we compare the improved classification model with several popular oil and gas layer classification models. These classification models are the standard RF model, SVM model, BSA-RF model, and DMSDL-BSA-RF model. Here, the RF classification model was first applied to the field of logging. In order to evaluate the performance of the recognition model, we select the following performance indicators:

(21) $$\begin{array}{c} \text{RMSE}=\sqrt{\frac{1}{N}\sum_{i=1}^{N}{\left(f_{i}-y_{i}\right)}^{2}},\\ \text{MAE}=\frac{1}{N}\sum_{i=1}^{N}\left|f_{i}-y_{i}\right|, \end{array}$$

where *y*_*i*_ and *f*_*i*_ are the classification output value and the expected output value, respectively.

RMSE is used to evaluate the accuracy of each classification model. MAE is used to show actual forecasting errors.  Table 15 records the performance indicator data of each classification model, and the best results are marked in bold. The smaller the RMSE and MAE, the better the classification model performs.

**Figure 6:**
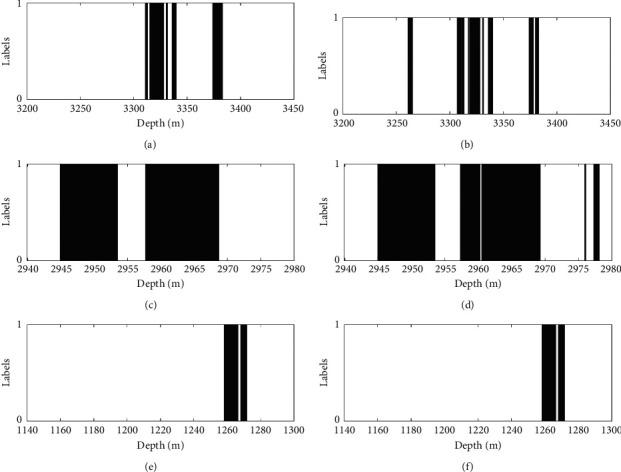
Classification of DMSDL-QBSA-RF: (a) the actual oil layer distribution of W1; (b) DMSDL-QBSA-RF oil layer distribution of W1; (c) the actual oil layer distribution of W2; (d) DMSDL-QBSA-RF oil layer distribution of 2; (e) the actual oil layer distribution of 3; (f) DMSDL-QBSA-RF oil layer distribution of 3.

From the performance indicator data of each classification model in Table 15, we can see that the DMSDL-QBSA-RF classification model can get the best recognition accuracy and all the accuracies are up to 90%. The recognition accuracy of the proposed classification model for W3 is up to 99.73%, and it has superior performance for oil and gas layer classification in other performance indicators and different wells. Secondly, DMSDL-QBSA can improve the performance of RF, and the parameters found by DMSDL-QBSA used in the RF classification model can improve the classification accuracy and keep running speed relatively fast at the same time. For example, the running times of DMSDL-QBSA-RF classification model for W1 and W2 are, respectively, 0.0504 seconds and 1.9292 seconds faster than the original RF classification model. Based on above results of data, the proposed classification model is better than the traditional RF and SVM model in oil layer classification. The comparison of oil layer classification result is shown in Figure 6, where, (a), (c), and (e) represent the actual oil layer distribution and (b), (d), and (f) represent DMSDL-QBSA-RF oil layer distribution. In addition, 0 means this depth has no oil or gas and 1 means this depth has oil or gas.

From Figure 6, we can see that the DMSDL-QBSA-RF classification model identifies that the oil layer distribution results are not much different from the test oil test results. It can accurately identify the distribution of oil and gas in a well. The DMSDL-QBSA-RF model is suitable for petroleum logging applications, which greatly reduces the difficulty of oil exploration and has a good application foreground.

## 5. Conclusion

This paper presents an improved BSA called DMSDL-QBSA, which employed the dynamic multi-swarm method, differential evolution, and quantum behavior to enhance the global and the local exploration capabilities of original BSA. First, 18 classical benchmark functions are used to verify the effectiveness of the improved method. The experimental study of the effects of these three strategies on the performance of DMSDL-QBSA revealed that the hybrid method has an excellent influence to improve the improvement of original GOA and especially original DE. Second, compared with the popular intelligence algorithms, such as GWO, WOA, SCA, GOA, and SSA, the DMSDL-QBSA can provide more competitive results on the 18 classical benchmark functions. Additionally, 30 complex CEC2014 test functions are used to better verify the performance of DMSDL-QBSA in a more comprehensively manner. The DMSDL-QBSA can show more excellent performance on the 18 classical benchmark functions. Finally, the improved DMSDL-QBSA is used to optimize the parameters of RF. Experimental results on actual oil logging prediction problem have proved that the classification accuracy of the established DMSDL-QBSA-RF classification model can get 94.00%, 94.24%, and 99.73% on these wells, and the accuracy is much higher than the original RF model. At the same time, the running speed performed faster than other four advanced classification models on most wells.

Although the proposed DMSDL-QBSA has been proven to be effective in solving general optimization problems, DMSDL-QBSA has some shortcomings that warrant further investigation. And in DMSDL-QBSA, due to the hybrid of three strategies, DMSDL-QBSA has needed more time than the classical BSA. Therefore, deploying the proposed algorithm to increase recognition efficiency is a worthwhile direction. In the future research work, the method presented in this paper can also be extended to solving discrete optimization problems and multiobjective optimization problems. Furthermore, applying the proposed DMSDL-QBSA-RF model to other fields such as financial prediction and biomedical science diagnosis is also an interesting future work.

## Figures and Tables

**Algorithm 1:**
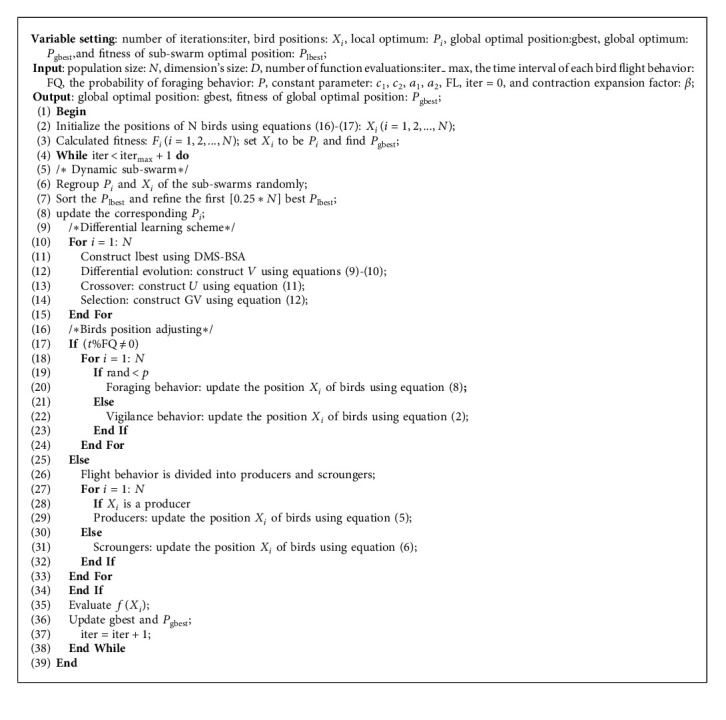
1: DMSDL-QBSA.

**Algorithm 2:**
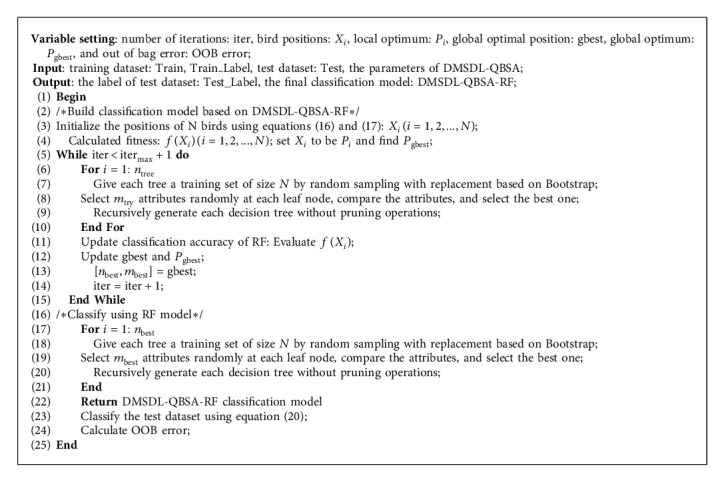
DMSDL-QBSA-RF classification model.

**Table 1:** 18 benchmark functions.

Name	Test function	Range
Sphere	*f*_1_(*x*)=∑_*i*=1_^*D*^*x*_*i*_^2^	[−100,100]^*D*^
Schwefel P2.22	*f*_2_=∑_*i*=1_^*D*^|*x*_*i*_|+∏_*i*=1_^*D*^|*x*_*i*_|	[−10,10]^*D*^
Schwefel P1.2	*f*_3_(*x*)=∑_*i*=1_^*D*^(∑_*j*_^*i*^*x*_*j*_)^2^	[−100,100]^*D*^
Generalized Rosenbrock	*f*_4_(*x*)=∑_*i*=1_^*D*−1^[100(*x*_*i*_^2^ − *x*_*i*+1_)^2^+(*x*_*i*_ − 1)^2^]	[−100,100]^*D*^
Step	*f*_5_(*x*)=∑_*i*=1_^*D*^(|*x*_*i*_+0.5|)^2^	[−100,100]^*D*^
Noise	*f*_6_(*x*)=∑_*i*=1_^*D*^*ix*_*i*_^4^+rand[0,1)	[−1.28, 1.28]^*D*^
SumSquares	*f*_7_(*x*)=∑_*i*=1_^*D*^*ix*_*i*_^2^	[−10,10]^*D*^
Zakharov	*f*_8_(*x*)=∑_*i*=1_^*D*^*x*_*i*_^2^+(∑_*i*=1_^*D*^0.5*ix*_*i*_)^2^+(∑_*i*=1_^*D*^0.5*ix*_*i*_)^4^	[−10,10]^*D*^
Schaffer	*f*_9_(*x*, *y*)=0.5+(sin^2^(*x*^2^ − *y*^2^) − 0.5/[1+0.001(*x*^2^ − *y*^2^)]^2^)	[−10,10]^*D*^

Generalized Schwefel 2.26	$f_{10}\left(x\right)=418.9829\;D-\sum_{i=1}^{D}\left(x\;\sin\sqrt{\left|x_{i}\right|}\right)$	[−500,500]^*D*^
Generalized Rastrigin	*f*_11_(*x*)=∑_*i*=1_^*D*^[*x*_*i*_^2^ − 10 cos(2*πx*_*i*_)+10]	[−5.12, 5.12]^*D*^
Ackley	$f_{12}=20+e-20\;\exp\left(-0.2\sqrt{\left(1/D\right)\sum_{i=1}^{D}x_{i}^{2}}\right)-\exp\left(\left(1/D\right)\sum_{i=1}^{D}\cos\;2\;\pi x_{i}\right)$	[−32,32]^*D*^
Generalized Griewank	$f_{13}\left(x\right)=\sum_{i=1}^{D}\left(x_{i}^{2}/4000\right)-\prod_{i=1}^{D}\cos\left(x_{i}/\sqrt{i}\right)+1$	[−600,600]^*D*^
Generalized Penalized 1	*f*_14_(*x*)=(*π*/*D*){10 sin^2^(*πy*_1_)+∑_*i*=1_^*D*−1^(*y*_*i*_ − 1)^2^[1+10 sin^2^(*πy*_*i*+1_)]+(*y*_*D*_ − 1)^2^}+∑_*i*=1_^*D*^*u*(*x*_*i*_, 10,100,4). $u\left(x_{i},a,k,m\right)=\left\{\begin{array}{cc} k{\left(x_{i}-a\right)}^{m}, & x_{i}>a\\ 0, & -a\leq x_{i}\leq a\\ k{\left(-x_{i}-a\right)}^{m}, & x_{i}<-a \end{array}\right.$_,_*y*_*i*_=1+(1/4)*x*_*i*_	[−50,50]^*D*^
Generalized Penalized 2	*f*_15_(*x*)=(1/10){10 sin^2^(3*πx*_1_)+∑_*i*=1_^*D*−1^(*x*_*i*_ − 1)^2^[1+ sin^2^(3*πx*_*i*+1_)]+(*x*_*D*_ − 1)^2^[1+ sin^2^(2*πx*_*D*_)]}+∑_*i*=1_^*D*^*u*(*x*_*i*_, 5,100,4)	[−50,50]^*D*^
Alpine	*f*_16_(*x*)=∑_*i*=1_^*D*^|*x*_*i*_sin *x*_*i*_+0.1*x*_*i*_|	[−10,10]^*D*^
Booth	*f*_17_(*x*)=(*x*_1_+2*x*_2_ − 7)^2^+(2*x*_1_+*x*_2_ − 5)^2^	[−10,10]^*D*^
Levy	*f*_18_(*x*)= sin^2^(*πx*_1_)+∑_*i*=1_^*D*^{(*y*_*i*_ − 1)^2^[1+10 sin^2^(*πy*_*i*_+1)]}+(*y*_*i*_ − 1)^2^[1+10 sin^2^(2*πy*_*D*_)]	[−10,10]^*D*^

**Table 2:** Summary of the CEC′14 test functions.

Type	No.	Functions	Fi^∗^ = Fi (*x*^∗^)
Unimodal functions	*F*_1_	Rotated High-Conditioned Elliptic Function	100
	*F*_2_	Rotated Bent Cigar Function	200
	*F*_3_	Rotated Discus Function	300

Simple multimodal functions	*F*_4_	Shifted and Rotated Rosenbrock's Function	400
	*F*_5_	Shifted and Rotated Ackley's Function	500
	*F*_6_	Shifted and Rotated Weierstrass Function	600
	*F*_7_	Shifted and Rotated Griewank's Function	700
	*F*_8_	Shifted Rastrigin's Function	800
	*F*_9_	Shifted and Rotated Rastrigin's Function	900
	*F*_10_	Shifted Schwefel's Function	1000
	*F*_11_	Shifted and Rotated Schwefel's Function	1100
	*F*_12_	Shifted and Rotated Katsuura Function	1200
	*F*_13_	Shifted and Rotated HappyCat function	1300
	*F*_14_	Shifted and Rotated HGBat Function	1400
	*F*_15_	Shifted and Rotated Expanded Griewank's Plus Rosenbrock's Function	1500
	*F*_16_	Shifted and Rotated Expanded Scaffer's F6 Function	1600

Hybrid functions	*F*_17_	Hybrid function 1 (*N* = 3)	1700
	*F*_18_	Hybrid function 2 (*N* = 3)	1800
	*F*_19_	Hybrid function 3 (*N* = 4)	1900
	*F*_20_	Hybrid function 4 (*N* = 4)	2000
	*F*_21_	Hybrid function 5 (*N* = 5)	2100
	*F*_22_	Hybrid function 6 (*N* = 5)	2200

Composition functions	*F*_23_	Composition function 1 (*N* = 5)	2300
	*F*_24_	Composition function 2 (*N* = 3)	2400
	*F*_25_	Composition function 3 (*N* = 3)	2500
	*F*_26_	Composition function 4 (*N* = 5)	2600
	*F*_27_	Composition function 5 (*N* = 5)	2700
	*F*_28_	Composition function 6 (*N* = 5)	2800
	*F*_29_	Composition function 7 (*N* = 3)	2900
	*F*_30_	Composition function 8 (*N* = 3)	3000

Search range: [−100,100]^*D*^			

**Table 3:** Parameter settings.

Algorithm	Parameter settings
BSA	*c*_1_=*c*_2_=1.5, *a*_1_=*a*_2_=1, FQ=10
DE	*F*=0.5, CR=0.1
DMSDL-PSO	*c*_1_=*c*_2_=1.49445, *F*=0.5, CR=0.1, sub-swarm = 10
DMSDL-BSA	*c*_1_=*c*_2_=1.5, *a*_1_=*a*_2_=1, FQ=10, *F*=0.5, CR=0.1, sub-swarm = 10
DMSDL-QBSA	*c*_1_=*c*_2_=1.5, *a*_1_=*a*_2_=1, FQ=10, *F*=0.5, CR=0.1, sub-swarm = 10

**Table 4:** Comparison on nine unimodal functions with 5 hybrid algorithms (Dim = 10).

Function	Term	BSA	DE	DMSDL-PSO	DMSDL-BSA	DMSDL-QBSA
*f*_1_	Max	1.0317*E* + 04	1.2466*E* + 04	1.7232*E* + 04	1.0874*E* + 04	**7.4436*E*** + **01**
	Min	**0**	4.0214*E* + 03	1.6580*E* − 02	**0**	**0**
	Mean	6.6202*E* + 00	4.6622*E* + 03	6.0643*E* + 01	6.3444*E* + 00	**3.3920*E*** − **02**
	Var	2.0892*E* + 02	8.2293*E* + 02	7.3868*E* + 02	1.9784*E* + 02	**1.2343*E*** + **00**

*f*_2_	Max	3.3278*E* + 02	1.8153*E* + 02	8.0436*E*+01	4.9554*E*+02	**2.9845*E*** + **01**
	Min	4.9286*E* − 182	1.5889*E* + 01	1.0536*E* − 01	3.0074*E* − 182	**0**
	Mean	5.7340*E* − 02	1.8349*E* + 01	2.9779*E* + 00	6.9700*E* − 02	**1.1220*E*** − **02**
	Var	3.5768*E* + 00	4.8296*E* + 00	2.4966*E* + 00	5.1243*E* + 00	**4.2864*E*** − **01**

*f*_3_	Max	1.3078*E* + 04	1.3949*E* + 04	1.9382*E* + 04	1.2899*E* + 04	**8.4935*E*** + **01**
	Min	3.4873*E* − 250	4.0327*E* + 03	6.5860*E* − 02	1.6352*E* − 249	**0**
	Mean	7.6735*E* + 00	4.6130*E* + 03	8.6149*E* + 01	7.5623*E* + 00	**3.3260*E*** − **02**
	Var	2.4929*E* + 02	8.4876*E* + 02	8.1698*E* + 02	2.4169*E* + 02	**1.2827*E*** + **00**

*f*_4_	Max	2.5311*E* + 09	2.3900*E* + 09	4.8639*E* + 09	3.7041*E* + 09	**3.5739*E*** + **08**
	Min	5.2310*E* + 00	2.7690*E* + 08	8.4802*E* + 00	**5.0021*E*** + **00**	8.9799*E* + 00
	Mean	6.9192*E* + 05	3.3334*E* + 08	1.1841*E* + 07	9.9162*E* + 05	**6.8518*E*** + **04**
	Var	3.6005*E* + 07	1.4428*E* + 08	1.8149*E* + 08	4.5261*E* + 07	**4.0563*E*** + **06**

*f*_5_	Max	1.1619*E* + 04	1.3773*E* + 04	1.6188*E* + 04	1.3194*E* + 04	**5.1960*E*** + **03**
	Min	5.5043*E* − 15	5.6109*E* + 03	1.1894*E* − 02	**4.2090*E*** − **15**	1.5157*E* + 00
	Mean	5.9547*E* + 00	6.3278*E* + 03	5.2064*E* + 01	6.5198*E* + 00	**3.5440*E*** + **00**
	Var	2.0533*E* + 02	9.6605*E* + 02	6.2095*E* + 02	2.2457*E* + 02	**7.8983*E*** + **01**

*f*_6_	Max	3.2456*E* + 00	7.3566*E* + 00	8.9320*E* + 00	2.8822*E* + 00	**1.4244*E*** + **00**
	Min	1.3994*E* − 04	1.2186*E* + 00	2.2482*E* − 03	8.2911*E* − 05	**1.0911*E*** − **05**
	Mean	2.1509*E* − 03	1.4021*E* + 00	1.1982*E* − 01	1.9200*E* − 03	**6.1476*E*** − **04**
	Var	5.3780*E* − 02	3.8482*E* − 01	3.5554*E* − 01	5.0940*E* − 02	**1.9880*E*** − **02**

*f*_7_	Max	4.7215*E* + 02	6.7534*E* + 02	5.6753*E* + 02	5.3090*E* + 02	**2.3468*E*** + **02**
	Min	**0**	2.2001*E* + 02	5.6300*E* − 02	**0**	**0**
	Mean	2.4908*E* − 01	2.3377*E* + 02	9.2909*E* + 00	3.0558*E* − 01	**9.4500*E*** − **02**
	Var	8.5433*E* + 00	3.3856*E* + 01	2.2424*E* + 01	1.0089*E* + 01	**3.5569*E*** + **00**

*f*_8_	Max	3.2500*E* + 02	2.4690*E* + 02	2.7226*E* + 02	2.8001*E* + 02	**1.7249*E*** + **02**
	Min	1.4678*E* − 239	8.3483*E* + 01	5.9820*E* − 02	8.9624*E* − 239	**0**
	Mean	1.9072*E* − 01	9.1050*E* + 01	7.9923*E* + 00	2.3232*E* − 01	**8.1580*E*** − **02**
	Var	6.3211*E* + 00	1.3811*E* + 01	1.7349*E* + 01	6.4400*E* + 00	**2.9531*E*** + **00**

**Table 5:** Comparison on nine unimodal functions with hybrid algorithms (Dim = 2).

Function	Term	BSA	DE	DMSDL-PSO	DMSDL-BSA	DMSDL-QBSA
*f*_1_	Max	1.8411*E* + 02	3.4713*E* + 02	2.9347*E* + 02	1.6918*E* + 02	**1.6789*E*** + **00**
	Min	**0**	5.7879*E* − 01	**0**	**0**	3.2988*E* − 238
	Mean	5.5890*E* − 02	1.1150*E* + 00	1.6095*E* − 01	3.6580*E* − 02	**4.2590*E*** − **03**
	Var	2.6628*E* + 00	5.7218*E* + 00	5.4561*E* + 00	2.0867*E* + 00	**7.5858*E*** − **02**

*f*_2_	Max	2.2980*E* + 00	2.1935*E* + 00	3.2363*E* + 00	3.1492*E* + 00	**1.1020*E*** + **00**
	Min	5.5923*E* − 266	8.2690*E* − 02	2.9096*E* − 242	3.4367*E* − 241	**0**
	Mean	9.2769*E* − 04	9.3960*E* − 02	7.4900*E* − 03	1.2045*E* − 03	**2.1565*E − *04**
	Var	3.3310*E* − 02	6.9080*E* − 02	4.0100*E* − 02	4.2190*E* − 02	**1.3130*E − *02**

*f*_3_	Max	**1.0647*E*** + **02**	1.3245*E* + 02	3.6203*E* + 02	2.3793*E* + 02	1.3089*E* + 02
	Min	**0**	5.9950*E* − 01	**0**	**0**	**0**
	Mean	2.3040*E* − 02	8.5959*E* − 01	2.5020*E* − 01	6.3560*E* − 02	**1.7170*E − *02**
	Var	**1.2892*E*** + **00**	2.8747*E* + 00	7.5569*E* + 00	2.9203*E* + 00	1.3518*E* + 00

*f*_4_	Max	1.7097*E* + 02	6.1375*E* + 01	6.8210*E* + 01	5.9141*E* + 01	**1.4726*E*** + **01**
	Min	1.6325*E* − 21	4.0940*E* − 02	8.2726*E* − 13	3.4830*E* − 25	**0**
	Mean	2.2480*E* − 02	9.3940*E* − 02	1.5730*E* − 02	**1.1020*E − *02**	1.4308*E* − 02
	Var	1.7987*E* + 00	7.4859*E* − 01	7.6015*E* − 01	6.4984*E* − 01	**2.7598*E − *01**

*f*_5_	Max	1.5719*E* + 02	2.2513*E* + 02	3.3938*E* + 02	1.8946*E* + 02	**8.7078*E*** + **01**
	Min	**0**	7.0367*E* − 01	**0**	**0**	**0**
	Mean	3.4380*E* − 02	1.8850*E* + 00	1.7082*E* − 01	5.0090*E* − 02	**1.1880*E − *02**
	Var	1.9018*E* + 00	5.6163*E* + 00	5.9868*E* + 00	2.4994*E* + 00	**9.1749*E − *01**

*f*_6_	Max	1.5887*E* − 01	1.5649*E* − 01	1.5919*E* − 01	1.3461*E* − 01	**1.0139*E − *01**
	Min	2.5412*E* − 05	4.5060*E* − 04	5.9140*E* − 05	4.1588*E* − 05	**7.3524*E − *06**
	Mean	2.3437*E* − 04	1.3328*E* − 03	6.0989*E* − 04	2.3462*E* − 04	**9.2394*E − *05**
	Var	2.4301*E* − 03	3.6700*E* − 03	3.5200*E* − 03	1.9117*E* − 03	**1.4664*E − *03**

*f*_7_	Max	3.5804*E* + 00	2.8236*E* + 00	**1.7372*E*** + **00**	2.7513*E* + 00	1.9411*E* + 00
	Min	**0**	7.6633*E* − 03	**0**	**0**	**0**
	Mean	8.5474*E* − 04	1.6590*E* − 02	8.6701*E* − 04	7.6781*E* − 04	**3.1439*E − *04**
	Var	4.4630*E* − 02	6.0390*E* − 02	2.4090*E* − 02	3.7520*E* − 02	**2.2333*E − *02**

*f*_8_	Max	4.3247*E* + 00	2.1924*E* + 00	5.3555*E* + 00	3.3944*E* + 00	**5.5079*E − *01**
	Min	**0**	8.6132*E* − 03	**0**	**0**	**0**
	Mean	1.1649*E* − 03	1.9330*E* − 02	1.7145*E* − 03	7.3418*E* − 04	**6.9138*E − *05**
	Var	5.9280*E* − 02	4.7800*E* − 02	7.5810*E* − 02	4.1414*E* − 02	**5.7489*E − *03**

*f*_9_	Max	2.7030*E* − 02	3.5200*E* − 02	1.7240*E* − 02	4.0480*E* − 02	**2.5230*E − *02**
	Min	**0**	5.0732*E* − 03	**0**	**0**	**0**
	Mean	6.1701*E* − 05	6.2500*E* − 03	8.8947*E* − 04	8.4870*E* − 05	**2.7362*E − *05**
	Var	7.6990*E* − 04	1.3062*E* − 03	2.0400*E* − 03	9.6160*E* − 04	**5.5610*E − *04**

**Table 6:** Comparison on nine multimodal functions with hybrid algorithms (Dim = 10).

Function	Term	BSA	DE	DMSDL-PSO	DMSDL-BSA	DMSDL-QBSA
*f*_10_	Max	2.8498*E* + 03	2.8226*E* + 03	3.0564*E* + 03	**2.7739*E*** + **03**	2.8795*E* + 03
	Min	**1.2553*E*** + **02**	1.8214*E* + 03	1.2922*E* + 03	1.6446*E* + 02	1.1634*E* + 03
	Mean	**2.5861*E*** + **02**	1.9229*E* + 03	1.3185*E* + 03	3.1119*E* + 02	1.2729*E* + 03
	Var	2.4093*E* + 02	**1.2066*E*** + **02**	1.2663*E* + 02	2.5060*E* + 02	1.2998*E* + 02

*f*_11_	Max	1.2550*E* + 02	1.0899*E* + 02	1.1806*E* + 02	1.1243*E* + 02	**9.1376*E*** + **01**
	Min	**0**	6.3502*E* + 01	1.0751*E* + 01	**0**	**0**
	Mean	2.0417*E* − 01	6.7394*E* + 01	3.9864*E* + 01	1.3732*E* − 01	**6.8060*E*** − **02**
	Var	3.5886*E* + 00	5.8621*E* + 00	1.3570*E* + 01	3.0325*E* + 00	**2.0567*E*** + **00**

*f*_12_	Max	2.0021*E* + 01	1.9910*E* + 01	1.9748*E* + 01	1.9254*E* + 01	**1.8118*E*** + **01**
	Min	**8.8818*E*** − **16**	1.6575*E* + 01	7.1700*E* − 02	**8.8818*E*** − **16**	**8.8818*E*** − **16**
	Mean	3.0500*E* − 02	1.7157*E* + 01	3.0367*E* + 00	3.8520*E* − 02	**1.3420*E*** − **02**
	Var	5.8820*E* − 01	5.2968*E* − 01	1.6585*E* + 00	6.4822*E* − 01	**4.2888*E*** − **01**

*f*_13_	Max	1.0431*E* + 02	1.3266*E* + 02	1.5115*E* + 02	1.2017*E* + 02	**6.1996*E*** + **01**
	Min	**0**	4.5742*E* + 01	2.1198*E* − 01	**0**	**0**
	Mean	6.1050*E* − 02	5.2056*E* + 01	3.0613*E* + 00	6.9340*E* − 02	**2.9700*E − *02**
	Var	1.8258*E* + 00	8.3141*E* + 00	1.5058*E* + 01	2.2452*E* + 00	**1.0425*E*** + **00**

*f*_14_	Max	**8.4576*E*** + **06**	3.0442*E* + 07	5.3508*E* + 07	6.2509*E* + 07	8.5231*E* + 06
	Min	1.7658*E* − 13	1.9816*E* + 06	4.5685*E* − 05	**1.6961*E − *13**	5.1104*E − *01
	Mean	1.3266*E* + 03	3.1857*E* + 06	6.8165*E* + 04	8.8667*E* + 03	**1.1326*E*** + **03**
	Var	9.7405*E* + 04	1.4876*E* + 06	1.4622*E* + 06	6.4328*E* + 05	**8.7645*E*** + **04**

*f*_15_	Max	1.8310*E* + 08	1.4389*E* + 08	1.8502*E* + 08	1.4578*E* + 08	**2.5680*E*** + **07**
	Min	1.7942*E* − 11	1.0497*E* + 07	2.4500*E − *03	**1.1248*E − *11**	9.9870*E − *01
	Mean	3.7089*E* + 04	1.5974*E* + 07	2.0226*E* + 05	3.8852*E* + 04	**3.5739*E*** + **03**
	Var	2.0633*E* + 06	1.0724*E* + 07	4.6539*E* + 06	2.1133*E* + 06	**2.6488*E*** + **05**

*f*_16_	Max	1.3876*E* + 01	1.4988*E* + 01	1.4849*E* + 01	1.3506*E* + 01	**9.3280*E*** + **00**
	Min	4.2410*E* − 174	6.8743*E* + 00	2.5133*E* − 02	7.3524*E* − 176	**0**
	Mean	1.3633*E* − 02	7.2408*E* + 00	2.5045*E* + 00	1.3900*E* − 02	**5.1800*E − *03**
	Var	3.3567*E* − 01	7.7774*E* − 01	1.0219*E* + 00	3.4678*E* − 01	**1.7952*E − *01**

*f*_18_	Max	3.6704*E* + 01	3.6950*E* + 01	2.8458*E* + 01	2.6869*E* + 01	**2.4435*E*** + **01**
	Min	2.0914*E* − 11	9.7737*E* + 00	3.3997*E − *03	**5.9165*E*** − **12**	7.5806*E* − 01
	Mean	6.5733*E* − 02	1.2351*E* + 01	6.7478*E − *01	**5.6520*E*** − **02**	7.9392*E* − 01
	Var	6.7543*E* − 01	2.8057*E* + 00	1.4666*E* + 00	6.4874*E* − 01	**3.6928*E*** − **01**

**Table 7:** Comparison on nine multimodal functions with hybrid algorithms (Dim = 2).

Function	Term	BSA	DE	DMSDL-PSO	DMSDL-BSA	DMSDL-QBSA
*f*_10_	Max	**2.0101*E*** + **02**	2.8292*E* + 02	2.9899*E* + 02	2.4244*E* + 02	2.8533*E* + 02
	Min	**2.5455*E*** − **05**	3.7717*E* + 00	2.3690*E* + 01	**2.5455*E*** − **05**	9.4751*E* + 01
	Mean	**3.4882*E*** − **01**	8.0980*E* + 00	2.5222*E* + 01	4.2346*E − *01	9.4816*E* + 01
	Var	5.7922*E* + 00	1.0853*E* + 01	1.5533*E* + 01	5.6138*E* + 00	**2.8160*E*** + **00**

*f*_11_	Max	**4.7662*E*** + **00**	4.7784*E* + 00	1.1067*E* + 01	8.7792*E* + 00	8.1665*E* + 00
	Min	**0**	3.8174*E − *01	**0**	**0**	**0**
	Mean	**2.7200*E*** − **03**	6.0050*E − *01	3.1540*E − *02	4.2587*E − *03	3.7800*E − *03
	Var	**8.6860*E*** − **02**	3.1980*E − *01	2.4862*E − *01	1.2032*E − *01	1.3420*E − *01

*f*_12_	Max	9.6893*E* + 00	**8.1811*E*** + **00**	1.1635*E* + 01	9.1576*E* + 00	8.4720*E* + 00
	Min	**8.8818*E*** − **16**	5.1646*E − *01	**8.8818*E*** − **16**	**8.8818*E*** − **16**	**8.8818*E*** − **16**
	Mean	**9.5600*E*** − **03**	6.9734*E − *01	3.4540*E − *02	9.9600*E − *03	2.8548*E − *03
	Var	2.1936*E − *01	6.1050*E − *01	2.5816*E − *01	2.1556*E − *01	**1.1804*E*** − **01**

*f*_13_	Max	4.4609*E* + 00	4.9215*E* + 00	4.1160*E* + 00	1.9020*E* + 00	**1.6875*E*** + **00**
	Min	**0**	1.3718*E − *01	**0**	**0**	**0**
	Mean	1.9200*E − *03	1.7032*E − *01	1.8240*E − *02	1.4800*E − *03	**5.7618*E*** − **04**
	Var	6.6900*E − *02	1.3032*E − *01	1.8202*E − *01	3.3900*E − *02	**2.2360*E*** − **02**

*f*_14_	Max	1.0045*E* + 01	1.9266*E* + 03	1.9212*E* + 01	5.7939*E* + 02	**8.2650*E*** + **00**
	Min	**2.3558*E*** − **31**	1.3188*E − *01	**2.3558*E*** − **31**	**2.3558*E*** − **31**	**2.3558*E*** − **31**
	Mean	4.1600*E − *03	3.5402*E − *01	1.0840*E − *02	6.1420*E − *02	**1.3924*E − *03**
	Var	1.7174*E − *01	1.9427*E* + 01	3.9528*E − *01	5.8445*E* + 00	**8.7160*E − *02**

*f*_15_	Max	6.5797*E* + 04	4.4041*E* + 03	1.4412*E* + 05	8.6107*E* + 03	**2.6372*E*** + **00**
	Min	**1.3498*E − *31**	9.1580*E − *02	**1.3498*E − *31**	**1.3498*E − *31**	7.7800*E − *03
	Mean	7.1736*E* + 00	8.9370*E − *01	1.7440*E* + 01	9.0066*E − *01	**8.2551*E − *03**
	Var	6.7678*E* + 02	5.4800*E* + 01	1.4742*E* + 03	8.7683*E* + 01	**2.8820*E − *02**

*f*_16_	Max	6.2468*E − *01	6.4488*E − *01	5.1564*E − *01	8.4452*E − *01	**3.9560*E − *01**
	Min	6.9981*E − *08	2.5000*E − *03	1.5518*E − *240	2.7655*E − *07	**0**
	Mean	2.7062*E − *04	6.9400*E − *03	6.8555*E − *04	2.0497*E − *04	**6.1996*E − *05**
	Var	1.0380*E − *02	1.7520*E − *02	8.4600*E − *03	1.0140*E − *02	**4.4000*E − *03**

*f*_17_	Max	5.1946*E* + 00	3.6014*E* + 00	2.3463*E* + 00	6.9106*E* + 00	**1.2521*E*** + **00**
	Min	2.6445*E − *11	2.6739*E − *02	**0**	1.0855*E − *10	**0**
	Mean	1.9343*E − *03	5.1800*E − *02	1.2245*E − *03	2.8193*E − *03	**1.5138*E − *04**
	Var	7.3540*E − *02	1.2590*E − *01	4.1620*E − *02	1.1506*E − *01	**1.2699*E − *02**

*f*_18_	Max	5.0214*E − *01	**3.4034*E − *01**	4.1400*E − *01	3.7422*E − *01	4.0295*E − *01
	Min	**1.4998*E − *32**	1.9167*E − *03	**1.4998*E − *32**	**1.4998*E − *32**	**1.4998*E − *32**
	Mean	1.0967*E − *04	4.1000*E − *03	1.8998*E − *04	1.4147*E − *04	**6.0718*E − *05**
	Var	6.1800*E − *03	1.0500*E − *02	6.5200*E − *03	5.7200*E − *03	**4.4014*E − *03**

**Table 8:** Comparison on 8 unimodal functions with popular algorithms (Dim = 10, FEs = 100000).

Function	Term	GWO	WOA	SCA	GOA	SSA	DMSDL-QBSA
*f*_1_	Max	1.3396*E* + 04	1.4767*E* + 04	1.3310*E* + 04	2.0099*E* + 01	4.8745*E* + 03	**9.8570*E − *01**
	Min	**0**	**0**	4.2905*E − *293	8.6468*E − *17	**0**	**0**
	Mean	3.5990*E* + 00	4.7621*E* + 00	1.4014*E* + 02	7.0100*E − *02	4.5864*E* + 00	**1.0483*E − *04**
	Var	1.7645*E* + 02	2.0419*E* + 02	8.5054*E* + 02	4.4200*E − *01	1.4148*E* + 02	**9.8725*E − *03**

*f*_2_	Max	3.6021*E* + 02	2.5789*E* + 03	6.5027*E* + 01	9.3479*E* + 01	3.4359*E* + 01	**3.2313*E − *01**
	Min	**0**	**0**	9.8354*E − *192	2.8954*E − *03	2.4642*E − *181	**0**
	Mean	5.0667*E − *02	2.9480*E − *01	4.0760*E − *01	3.1406*E* + 00	1.7000*E − *02	**1.1278*E − *04**
	Var	3.7270*E* + 00	2.6091*E* + 01	2.2746*E* + 00	3.9264*E* + 00	5.4370*E − *01	**4.8000*E − *03**

*f*_3_	Max	1.8041*E* + 04	1.6789*E* + 04	2.4921*E* + 04	6.5697*E* + 03	1.1382*E* + 04	**4.0855*E*** + **01**
	Min	**0**	1.0581*E − *18	7.6116*E − *133	2.8796*E − *01	3.2956*E − *253	1.5918*E − *264
	Mean	7.4511*E* + 00	2.8838*E* + 02	4.0693*E* + 02	4.8472*E* + 02	9.2062*E* + 00	**4.8381*E − *03**
	Var	2.6124*E* + 02	1.4642*E* + 03	1.5913*E* + 03	7.1786*E* + 02	2.9107*E* + 02	**4.1383*E − *01**

*f*_4_	Max	2.1812*E* + 09	5.4706*E* + 09	8.4019*E* + 09	1.1942*E* + 09	4.9386*E* + 08	**7.5188*E*** + **01**
	Min	4.9125*E* + 00	3.5695*E* + 00	5.9559*E* + 00	2.2249*E* + 02	4.9806*E* + 00	**2.9279*E − *13**
	Mean	4.9592*E* + 05	2.4802*E* + 06	4.4489*E* + 07	5.0021*E* + 06	3.3374*E* + 05	**2.4033*E − *02**
	Var	2.9484*E* + 07	1.1616*E* + 08	4.5682*E* + 08	3.9698*E* + 07	1.1952*E* + 07	**9.7253*E − *01**

*f*_5_	Max	1.8222*E* + 04	1.5374*E* + 04	1.5874*E* + 04	1.2132*E* + 03	1.6361*E* + 04	**1.8007*E*** + **00**
	Min	1.1334*E − *08	8.3228*E − *09	2.3971*E − *01	2.7566*E − *10	2.6159*E − *16	**1.0272*E − *33**
	Mean	5.1332*E* + 00	5.9967*E* + 00	1.2620*E* + 02	5.8321*E* + 01	8.8985*E* + 00	**2.3963*E − *04**
	Var	2.3617*E* + 02	2.3285*E* + 02	8.8155*E* + 02	1.0872*E* + 02	2.9986*E* + 02	**1.8500*E − *02**

*f*_6_	Max	7.4088*E* + 00	8.3047*E* + 00	8.8101*E* + 00	6.8900*E − *01	4.4298*E* + 00	**2.5787*E − *01**
	Min	1.8112*E − *05	3.9349*E − *05	4.8350*E − *05	8.9528*E − *02	4.0807*E − *05	1.0734*E − *04
	Mean	1.8333*E − *03	3.2667*E − *03	4.8400*E − *02	9.3300*E − *02	2.1000*E − *03	**5.2825*E − *04**
	Var	9.4267*E − *02	1.0077*E − *01	2.9410*E − *01	3.5900*E − *02	6.5500*E − *02	**4.6000*E − *03**

*f*_7_	Max	7.3626*E* + 02	6.8488*E* + 02	8.0796*E* + 02	3.9241*E* + 02	8.2036*E* + 02	**1.4770*E*** + **01**
	Min	**0**	**0**	1.9441*E − *292	7.9956*E − *07	**0**	**0**
	Mean	2.0490*E − *01	2.8060*E − *01	4.9889*E* + 00	1.6572*E* + 01	2.7290*E − *01	**1.8081*E − *03**
	Var	9.5155*E* + 00	1.0152*E* + 01	3.5531*E* + 01	2.3058*E* + 01	1.0581*E* + 01	**1.6033*E − *01**

*f*_8_	Max	1.2749*E* + 03	5.9740*E* + 02	3.2527*E* + 02	2.3425*E* + 02	2.0300*E* + 02	**2.0423*E*** + **01**
	Min	**0**	4.3596*E − *35	1.5241*E − *160	3.6588*E − *05	1.0239*E − *244	**0**
	Mean	3.1317*E − *01	1.0582*E* + 01	1.0457*E* + 01	1.2497*E* + 01	2.1870*E − *01	**2.6947*E − *03**
	Var	1.4416*E* + 01	4.3485*E* + 01	3.5021*E* + 01	2.5766*E* + 01	6.2362*E* + 00	**2.1290*E − *01**

**Table 9:** Comparison on 8 multimodal functions with popular algorithms (Dim = 10, FEs = 100000).

Function	Term	GWO	WOA	SCA	GOA	SSA	DMSDL-QBSA
*f*_10_	Max	2.9544*E* + 03	2.9903*E* + 03	2.7629*E* + 03	2.9445*E* + 03	3.2180*E* + 03	3.0032*E* + 03
	Min	1.3037*E* + 03	8.6892*E − *04	1.5713*E* + 03	1.3438*E* + 03	**1.2839*E − *04**	1.2922*E* + 03
	Mean	1.6053*E* + 03	1.4339*E* + 01	1.7860*E* + 03	1.8562*E* + 03	**2.5055*E*** + **02**	1.2960*E* + 03
	Var	2.6594*E* + 02	1.2243*E* + 02	1.6564*E* + 02	5.1605*E* + 02	3.3099*E* + 02	**5.8691*E*** + **01**

*f*_11_	Max	1.3792*E* + 02	1.2293*E* + 02	1.2313*E* + 02	1.1249*E* + 02	3.2180*E* + 03	**1.8455*E*** + **01**
	Min	**0**	**0**	**0**	1.2437*E* + 01	1.2839*E − *04	**0**
	Mean	4.4220*E − *01	1.1252*E* + 00	9.9316*E* + 00	2.8378*E* + 01	2.5055*E* + 02	**3.2676*E − *03**
	Var	4.3784*E* + 00	6.5162*E* + 00	1.9180*E* + 01	1.6240*E* + 01	3.3099*E* + 02	**1.9867*E − *01**

*f*_12_	Max	2.0257*E* + 01	2.0043*E* + 01	1.9440*E* + 01	1.6623*E* + 01	3.2180*E* + 03	**1.9113*E*** + **00**
	Min	4.4409*E − *15	3.2567*E − *15	3.2567*E − *15	2.3168*E* + 00	1.2839*E − *04	**8.8818*E − *16**
	Mean	1.7200*E − *02	4.2200*E − *02	8.8870*E − *01	5.5339*E* + 00	2.5055*E* + 02	**3.1275*E − *04**
	Var	4.7080*E − *01	6.4937*E − *01	3.0887*E* + 00	2.8866*E* + 00	3.3099*E* + 02	**2.2433*E − *02**

*f*_13_	Max	1.5246*E* + 02	1.6106*E* + 02	1.1187*E* + 02	6.1505*E* + 01	3.2180*E* + 03	**3.1560*E − *01**
	Min	3.3000*E − *03	**0**	**0**	2.4147*E − *01	1.2839*E − *04	**0**
	Mean	4.6733*E − *02	9.3867*E − *02	1.2094*E* + 00	3.7540*E* + 00	2.5055*E* + 02	**3.3660*E − *05**
	Var	1.9297*E* + 00	2.6570*E* + 00	6.8476*E* + 00	4.1936*E* + 00	3.3099*E* + 02	**3.1721*E − *03**

*f*_14_	Max	9.5993*E* + 07	9.9026*E* + 07	5.9355*E* + 07	6.1674 *E* + 06	3.2180*E* + 03	**6.4903*E − *01**
	Min	3.8394*E − *09	1.1749*E − *08	9.6787*E − *03	1.8099*E − *04	1.2839*E − *04	**4.7116*E − *32**
	Mean	1.2033*E* + 04	3.5007*E* + 04	4.8303*E* + 05	1.0465*E* + 04	2.5055*E* + 02	**8.9321*E − *05**
	Var	9.8272*E* + 05	1.5889*E* + 06	4.0068*E* + 06	1.9887*E* + 05	3.3099*E* + 02	**6.8667*E − *03**

*f*_15_	Max	2.2691*E* + 08	2.4717*E* + 08	1.1346*E* + 08	2.8101*E* + 07	3.2180*E* + 03	**1.6407*E − *01**
	Min	3.2467*E − *02	4.5345*E − *08	1.1922*E − *01	3.5465*E − *05	1.2839*E − *04	**1.3498*E − *32**
	Mean	2.9011*E* + 04	4.3873*E* + 04	6.5529*E* + 05	7.2504*E* + 04	2.5055*E* + 02	**6.7357*E − *05**
	Var	2.3526*E* + 06	2.7453*E* + 06	7.1864*E* + 06	1.2814*E* + 06	3.3099*E* + 02	**2.4333*E − *03**

*f*_16_	Max	1.7692*E* + 01	1.7142*E* + 01	1.6087*E* + 01	8.7570*E* + 00	3.2180*E* + 03	**1.0959*E*** + **00**
	Min	2.6210*E − *07	0.0000*E* + 00	6.2663*E − *155	1.0497*E − *02	1.2839*E − *04	**0**
	Mean	1.0133*E − *02	3.9073*E − *01	5.9003*E − *01	2.4770*E* + 00	2.5055*E* + 02	**1.5200*E − *04**
	Var	3.0110*E − *01	9.6267*E − *01	1.4701*E* + 00	1.9985*E* + 00	3.3099*E* + 02	**1.1633*E − *02**

*f*_18_	Max	4.4776*E* + 01	4.3588*E* + 01	3.9095*E* + 01	1.7041*E* + 01	3.2180*E* + 03	**6.5613*E − *01**
	Min	1.9360*E − *01	9.4058*E − *08	2.2666*E − *01	3.9111*E* + 00	1.2839*E − *04	**1.4998*E − *32**
	Mean	2.1563*E − *01	4.7800*E − *02	9.8357*E − *01	5.4021*E* + 00	2.5055*E* + 02	**9.4518*E − *05**
	Var	5.8130*E − *01	1.0434*E* + 00	3.0643*E* + 00	1.6674*E* + 00	3.3099*E* + 02	**7.0251*E − *03**

**Table 10:** Comparison of numerical testing results on CEC2014 test sets (*F*1–*F*15, Dim = 10).

Function	Term	BSA	DE	DMSDL-PSO	DMSDL-BSA	DMSDL-QBSA
*F*_1_	Max	**2.7411*E*** + **08**	3.6316*E* + 09	6.8993*E* + 08	3.9664*E* + 08	9.6209*E* + 08
	Min	1.4794*E* + 07	3.1738*E* + 09	1.3205*E* + 06	**1.6929*E*** + **05**	1.7687*E* + 05
	Mean	3.1107*E* + 07	3.2020*E* + 09	6.7081*E* + 06	**1.3567*E*** + **06**	1.9320*E* + 06
	Var	1.2900*E* + 07	4.0848*E* + 07	2.6376*E* + 07	**1.0236*E*** + **07**	1.3093*E* + 07

*F*_2_	Max	**8.7763*E*** + **09**	1.3597*E* + 10	1.3515*E* + 10	1.6907*E* + 10	1.7326*E* + 10
	Min	1.7455*E* + 09	1.1878*E* + 10	3.6982*E* + 04	2.1268*E* + 05	**1.4074*E*** + **05**
	Mean	1.9206*E* + 09	1.1951*E* + 10	1.7449*E* + 08	5.9354*E* + 07	**4.8412*E*** + **07**
	Var	1.9900*E* + 08	**1.5615*E*** + **08**	9.2365*E* + 08	5.1642*E* + 08	4.2984*E* + 08

*F*_3_	Max	4.8974*E* + 05	**8.5863*E*** + **04**	2.6323*E* + 06	2.4742*E* + 06	5.3828*E* + 06
	Min	1.1067*E* + 04	1.4616*E* + 04	1.8878*E* + 03	1.2967*E* + 03	**6.3986*E*** + **02**
	Mean	1.3286*E* + 04	1.4976*E* + 04	1.0451*E* + 04	3.5184*E* + 03	**3.0848*E*** + **03**
	Var	6.5283*E* + 03	**1.9988*E*** + **03**	4.5736*E* + 04	4.1580*E* + 04	8.0572*E* + 04

*F*_4_	Max	**3.5252*E*** + **03**	9.7330*E* + 03	4.5679*E* + 03	4.9730*E* + 03	4.3338*E* + 03
	Min	5.0446*E* + 02	8.4482*E* + 03	**4.0222*E*** + **02**	4.0843*E* + 02	4.1267*E* + 02
	Mean	5.8061*E* + 02	8.5355*E* + 03	4.4199*E* + 02	4.3908*E* + 02	**4.3621*E*** + **02**
	Var	1.4590*E* + 02	1.3305*E* + 02	1.6766*E* + 02	1.2212*E* + 02	**8.5548*E*** + **01**

*F*_5_	Max	5.2111*E* + 02	5.2106*E* + 02	**5.2075*E*** + **02**	5.2098*E* + 02	5.2110*E* + 02
	Min	**5.2001*E*** + **02**	5.2038*E* + 02	5.2027*E* + 02	5.2006*E* + 02	5.2007*E* + 02
	Mean	**5.2003*E*** + **02**	5.2041*E* + 02	5.2033*E* + 02	5.2014*E* + 02	5.2014*E* + 02
	Var	7.8380*E − *02	5.7620*E − *02	**5.6433*E − *02**	1.0577*E − *01	9.9700*E − *02

*F*_6_	Max	**6.1243*E*** + **02**	6.1299*E* + 02	6.1374*E* + 02	6.1424*E* + 02	6.1569*E* + 02
	Min	6.0881*E* + 02	6.1157*E* + 02	6.0514*E* + 02	6.0288*E* + 02	**6.0257*E*** + **02**
	Mean	6.0904*E* + 02	6.1164*E* + 02	6.0604*E* + 02	6.0401*E* + 02	**6.0358*E*** + **02**
	Var	2.5608*E − *01	1.2632*E − *01	**1.0434*E*** + **00**	1.1717*E* + 00	1.3117*E* + 00

*F*_7_	Max	**8.7895*E*** + **02**	1.0459*E* + 03	9.1355*E* + 02	1.0029*E* + 03	9.3907*E* + 02
	Min	7.4203*E* + 02	1.0238*E* + 03	**7.0013*E*** + **02**	7.0081*E* + 02	7.0069*E* + 02
	Mean	7.4322*E* + 02	1.0253*E* + 03	7.1184*E* + 02	7.0332*E* + 02	**7.0290*E*** + **02**
	Var	4.3160*E* + 00	2.4258*E* + 00	2.8075*E* + 01	1.5135*E* + 01	**1.3815*E*** + **01**

*F*_8_	Max	**8.9972*E*** + **02**	9.1783*E* + 02	9.6259*E* + 02	9.2720*E* + 02	9.3391*E* + 02
	Min	8.4904*E* + 02	8.8172*E* + 02	8.3615*E* + 02	8.1042*E* + 02	**8.0877*E*** + **02**
	Mean	8.5087*E* + 02	8.8406*E* + 02	8.5213*E* + 02	8.1888*E* + 02	**8.1676*E*** + **02**
	Var	**2.1797*E*** + **00**	4.4494*E* + 00	8.6249*E* + 00	9.2595*E* + 00	9.7968*E* + 00

*F*_9_	Max	1.0082*E* + 03	**9.9538*E*** + **02**	1.0239*E* + 03	1.0146*E* + 03	1.0366*E* + 03
	Min	9.3598*E* + 02	9.6805*E* + 02	9.2725*E* + 02	9.2062*E* + 02	**9.1537*E*** + **02**
	Mean	9.3902*E* + 02	9.7034*E* + 02	9.4290*E* + 02	9.2754*E* + 02	**9.2335*E*** + **02**
	Var	3.4172*E* + 00	**3.2502*E*** + **00**	8.1321*E* + 00	9.0492*E* + 00	9.3860*E* + 00

*F*_10_	Max	**3.1087*E*** + **03**	3.5943*E* + 03	3.9105*E* + 03	3.9116*E* + 03	3.4795*E* + 03
	Min	2.2958*E* + 03	2.8792*E* + 03	1.5807*E* + 03	1.4802*E* + 03	**1.4316*E*** + **03**
	Mean	1.8668*E* + 03	2.9172*E* + 03	1.7627*E* + 03	1.6336*E* + 03	**1.6111*E*** + **03**
	Var	3.2703*E* + 01	6.1787*E* + 01	2.2359*E* + 02	**1.9535*E*** + **02**	2.2195*E* + 02

*F*_11_	Max	3.6641*E* + 03	**3.4628*E*** + **03**	4.0593*E* + 03	3.5263*E* + 03	3.7357*E* + 03
	Min	2.4726*E* + 03	2.8210*E* + 03	1.7855*E* + 03	1.4012*E* + 03	**1.2811*E*** + **03**
	Mean	2.5553*E* + 03	2.8614*E* + 03	1.8532*E* + 03	1.5790*E* + 03	**1.4869*E*** + **03**
	Var	8.4488*E* + 01	5.6351*E* + 01	**1.3201*E*** + **02**	2.1085*E* + 02	2.6682*E* + 02

*F*_12_	Max	1.2044*E* + 03	1.2051*E* + 03	1.2053*E* + 03	1.2055*E* + 03	**1.2044*E*** + **03**
	Min	1.2006*E* + 03	1.2014*E* + 03	1.2004*E* + 03	**1.2003*E*** + **03**	1.2017*E* + 03
	Mean	1.2007*E* + 03	1.2017*E* + 03	1.2007*E* + 03	**1.2003*E*** + **03**	1.2018*E* + 03
	Var	**1.4482*E − *01**	3.5583*E − *01	2.5873*E − *01	2.4643*E − *01	2.1603*E − *01

*F*_13_	Max	**1.3049*E*** + **03**	1.3072*E* + 03	1.3073*E* + 03	1.3056*E* + 03	1.3061*E* + 03
	Min	1.3005*E* + 03	1.3067*E* + 03	1.3009*E* + 03	**1.3003*E*** + **03**	1.3004*E* + 03
	Mean	1.3006*E* + 03	1.3068*E* + 03	1.3011*E* + 03	**1.3005*E*** + **03**	1.3006*E* + 03
	Var	3.2018*E − *01	5.0767*E − *02	4.2173*E − *01	3.6570*E − *01	**2.9913*E − *01**

*F*_14_	Max	**1.4395*E*** + **03**	1.4565*E* + 03	1.4775*E* + 03	1.4749*E* + 03	1.4493*E* + 03
	Min	1.4067*E* + 03	1.4522*E* + 03	1.4009*E* + 03	**1.4003*E*** + **03**	1.4005*E* + 03
	Mean	1.4079*E* + 03	1.4529*E* + 03	1.4024*E* + 03	**1.4008*E*** + **03**	1.4009*E* + 03
	Var	**2.1699*E*** + **00**	6.3013*E − *01	5.3198*E* + 00	3.2578*E* + 00	2.8527*E* + 00

*F*_15_	Max	5.4068*E* + 04	**3.3586*E*** + **04**	4.8370*E* + 05	4.0007*E* + 05	1.9050*E* + 05
	Min	1.9611*E* + 03	2.1347*E* + 04	1.5029*E* + 03	1.5027*E* + 03	**1.5026*E*** + **03**
	Mean	1.9933*E* + 03	2.2417*E* + 04	2.7920*E* + 03	1.5860*E* + 03	**1.5816*E*** + **03**
	Var	6.1622*E* + 02	**1.2832*E*** + **03**	1.7802*E* + 04	4.4091*E* + 03	2.7233*E* + 03

**Table 11:** Comparison of numerical testing results on CEC2014 test sets (*F*_16_– *F*_30_, Dim = 10).

Function	Term	BSA	DE	DMSDL-PSO	DMSDL-BSA	DMSDL-QBSA
*F*_16_	Max	1.6044*E* + 03	1.6044*E* + 03	1.6046*E* + 03	**1.6044*E*** + **03**	1.6046*E* + 03
	Min	1.6034*E* + 03	1.6041*E* + 03	1.6028*E* + 03	1.6021*E* + 03	**1.6019*E*** + **03**
	Mean	1.6034*E* + 03	1.6041*E* + 03	1.6032*E* + 03	1.6024*E* + 03	**1.6024*E*** + **03**
	Var	**3.3300*E − *02**	3.5900*E − *02	2.5510*E − *01	3.7540*E − *01	3.6883*E − *01

*F*_17_	Max	1.3711*E* + 07	**1.6481*E*** + **06**	3.3071*E* + 07	4.6525*E* + 07	8.4770*E* + 06
	Min	1.2526*E* + 05	4.7216*E* + 05	2.7261*E* + 03	2.0177*E* + 03	**2.0097*E*** + **03**
	Mean	1.6342*E* + 05	4.8499*E* + 05	8.7769*E* + 04	3.1084*E* + 04	**2.1095*E*** + **04**
	Var	2.0194*E* + 05	**2.9949*E*** + **04**	1.2284*E* + 06	8.7638*E* + 05	2.0329*E* + 05

*F*_18_	Max	7.7173*E* + 07	**3.5371*E*** + **07**	6.1684*E* + 08	1.4216*E* + 08	6.6050*E* + 08
	Min	4.1934*E* + 03	2.6103*E* + 06	2.2743*E* + 03	**1.8192*E*** + **03**	1.8288*E* + 03
	Mean	**1.6509*E*** + **04**	3.4523*E* + 06	1.2227*E* + 06	1.0781*E* + 05	1.9475*E* + 05
	Var	7.9108*E* + 05	**1.4888*E*** + **06**	2.1334*E* + 07	3.6818*E* + 06	1.0139*E* + 07

*F*_19_	Max	1.9851*E* + 03	2.5657*E* + 03	2.0875*E* + 03	1.9872*E* + 03	**1.9555*E*** + **03**
	Min	1.9292*E* + 03	2.4816*E* + 03	1.9027*E* + 03	**1.9023*E*** + **03**	1.9028*E* + 03
	Mean	1.9299*E* + 03	2.4834*E* + 03	1.9044*E* + 03	**1.9032*E*** + **03**	1.9036*E* + 03
	Var	**1.0820*E*** + **00**	3.8009*E* + 00	1.1111*E* + 01	3.3514*E* + 00	1.4209*E* + 00

*F*_20_	Max	**8.3021*E*** + **06**	2.0160*E* + 07	1.0350*E* + 07	6.1162*E* + 07	1.2708*E* + 08
	Min	5.6288*E* + 03	1.7838*E* + 06	2.1570*E* + 03	2.0408*E* + 03	**2.0241*E*** + **03**
	Mean	1.2260*E* + 04	1.8138*E* + 06	5.6957*E* + 03	**1.1337*E*** + **04**	4.5834*E* + 04
	Var	**1.0918*E*** + **05**	5.9134*E* + 05	1.3819*E* + 05	6.4167*E* + 05	2.1988*E* + 06

*F*_21_	Max	2.4495*E* + 06	1.7278*E* + 09	**2.0322*E*** + **06**	1.3473*E* + 07	2.6897*E* + 07
	Min	4.9016*E* + 03	**1.4049*E*** + **09**	3.3699*E* + 03	2.1842 *E* + 03	2.2314*E* + 03
	Mean	6.6613*E* + 03	1.4153*E* + 09	9.9472*E* + 03	1.3972*E* + 04	**7.4587*E*** + **03**
	Var	4.1702*E* + 04	2.2557*E* + 07	**3.3942*E*** + **04**	2.5098*E* + 05	2.8735*E* + 05

*F*_22_	Max	**2.8304*E*** + **03**	4.9894*E* + 03	3.1817*E* + 03	3.1865*E* + 03	3.2211*E* + 03
	Min	2.5070*E* + 03	4.1011*E* + 03	2.3492*E* + 03	2.2442*E* + 03	**2.2314*E*** + **03**
	Mean	**2.5081*E*** + **03**	4.1175*E* + 03	2.3694*E* + 03	2.2962*E* + 03	2.2687*E* + 03
	Var	5.4064*E* + 00	**3.8524*E*** + **01**	4.3029*E* + 01	4.8006*E* + 01	5.1234*E* + 01

*F*_23_	Max	2.8890*E* + 03	**2.6834*E*** + **03**	2.8758*E* + 03	3.0065*E* + 03	2.9923*E* + 03
	Min	2.5000*E* + 03	2.6031*E* + 03	**2.4870*E*** + **03**	2.5000*E* + 03	2.5000*E* + 03
	Mean	**2.5004*E*** + **03**	2.6088*E* + 03	2.5326*E* + 03	2.5010*E* + 03	2.5015*E* + 03
	Var	9.9486*E* + 00	8.6432*E* + 00	5.7045*E* + 01	**1.4213*E*** + **01**	1.7156*E* + 01

*F*_24_	Max	**2.6293*E*** + **03**	2.6074*E* + 03	2.6565*E* + 03	2.6491*E* + 03	2.6369*E* + 03
	Min	2.5816*E* + 03	2.6049*E* + 03	2.5557*E* + 03	**2.5246*E*** + **03**	2.5251*E* + 03
	Mean	2.5829*E* + 03	2.6052*E* + 03	2.5671*E* + 03	**2.5337*E*** + **03**	2.5338*E* + 03
	Var	1.3640*E* + 00	**3.3490*E − *01**	8.9434*E* + 00	1.3050*E* + 01	1.1715*E* + 01

*F*_25_	Max	2.7133*E* + 03	**2.7014*E*** + **03**	2.7445*E* + 03	2.7122*E* + 03	2.7327*E* + 03
	Min	2.6996*E* + 03	2.7003*E* + 03	2.6784*E* + 03	2.6789*E* + 03	**2.6635*E*** + **03**
	Mean	2.6996*E* + 03	2.7004*E* + 03	2.6831*E* + 03	**2.6903*E*** + **03**	2.6894*E* + 03
	Var	2.3283*E − *01	**9.9333*E − *02**	4.9609*E* + 00	7.1175*E* + 00	1.2571*E* + 01

*F*_26_	Max	**2.7056*E*** + **03**	2.8003*E* + 03	2.7058*E* + 03	2.7447*E* + 03	2.7116*E* + 03
	Min	2.7008*E* + 03	2.8000*E* + 03	2.7003*E* + 03	2.7002*E* + 03	**2.7002*E*** + **03**
	Mean	2.7010*E* + 03	2.8000*E* + 03	2.7005*E* + 03	2.7003*E* + 03	**2.7003*E*** + **03**
	Var	**3.1316*E − *01**	1.6500*E* − 02	3.9700*E* − 01	1.1168*E* + 00	3.5003*E* − 01

*F*_27_	Max	**3.4052*E*** + **03**	5.7614*E* + 03	3.3229*E* + 03	3.4188*E* + 03	3.4144*E* + 03
	Min	2.9000*E* + 03	3.9113*E* + 03	2.9698*E* + 03	2.8347*E* + 03	**2.7054*E*** + **03**
	Mean	2.9038*E* + 03	4.0351*E* + 03	2.9816*E* + 03	2.8668*E* + 03	**2.7219*E*** + **03**
	Var	**3.2449*E*** + **01**	2.0673*E* + 02	3.4400*E* + 01	6.2696*E* + 01	6.1325*E* + 01

*F*_28_	Max	4.4333*E* + 03	5.4138*E* + 03	4.5480*E* + 03	**4.3490*E*** + **03**	4.8154*E* + 03
	Min	3.0000*E* + 03	4.0092*E* + 03	3.4908*E* + 03	3.0000*E* + 03	**3.0000*E*** + **03**
	Mean	3.0079*E* + 03	4.0606*E* + 03	3.6004*E* + 03	**3.0063*E*** + **03**	3.0065*E* + 03
	Var	6.8101*E* + 01	9.2507*E* + 01	8.5705*E* + 01	**4.2080*E*** + **01**	5.3483*E* + 01

*F*_29_	Max	**2.5038*E*** + **07**	1.6181*E* + 08	5.9096*E* + 07	7.0928*E* + 07	6.4392*E* + 07
	Min	3.2066*E* + 03	1.0014*E* + 08	3.1755*E* + 03	**3.1005*E*** + **03**	3.3287*E* + 03
	Mean	5.7057*E* + 04	1.0476*E* + 08	8.5591*E* + 04	6.8388*E* + 04	**2.8663*E*** + **04**
	Var	**4.9839*E*** + **05**	6.7995*E* + 06	1.7272*E* + 06	1.5808*E* + 06	8.6740*E* + 05

*F*_30_	Max	**5.2623*E*** + **05**	2.8922*E* + 07	1.1938*E* + 06	1.2245*E* + 06	1.1393*E* + 06
	Min	5.9886*E* + 03	1.9017*E* + 07	3.7874*E* + 03	**3.6290*E*** + **03**	3.6416*E* + 03
	Mean	7.4148*E* + 03	2.0002*E* + 07	5.5468*E* + 03	4.3605*E* + 03	**4.1746*E*** + **03**
	Var	**1.1434*E*** + **04**	1.1968*E* + 06	3.2255*E* + 04	2.2987*E* + 04	1.8202*E* + 04

**Table 12:** Comparison of numerical testing results on eight UCI datasets.

Dataset	RF (%)	BSA-RF (%)	DMSDL-BSA-RF (%)	DMSDL-QBSA-RF (%)
Blood	75.40	80.80	79.46	**81.25**
Heart-statlog	82.10	**86.42**	**86.42**	**86.42**
Sonar	78.39	85.48	85.48	**88.71**
Appendicitis	83.23	87.10	90.32	**93.55**
Cleve	79.55	87.50	88.64	**89.77**
Magic	88.95	89.85	**90.25**	89.32
Mammographic	74.73	77.68	76.79	**79.46**
Australian	85.83	88.84	88.84	**90.78**

**Table 13:** Distribution of the logging data.

Well	Training dataset				Test dataset			
	Depth (m)	Data	Oil/gas layers	Dry layer	Depth (m)	Data	Oil/gas layers	Dry layer
*W*1	3027∼3058	250	203	47	3250∼3450	1600	237	1363
*W*2	2642∼2648	30	10	20	2940∼2978	191	99	92
*W*3	1040∼1059	160	47	113	1120∼1290	1114	96	1018

**Table 14:** Attribute reduction results of the logging dataset.

Well	Attributes	
*W*1	Actual attributes	{GR, DT, SP, WQ, LLD, LLS, DEN, NPHI, PE, U, TH, K, CALI}
	Reduction attributes	{GR, DT, SP, LLD, LLS, DEN, NPHI}

*W*2	Actual attributes	{DENSITY, GAMM, VCLOK, NEUTRO, PERM, POR, RESI, SONIC, SP, SW}
	Reduction attributes	{NEUTRO, PERM, POR, RESI, SW}

*W*3	Actual attributes	{AC, CNL, DEN, GR, RT, RI, RXO, SP, R2M, R025, BZSP, RA2, C1, C2, CALI, RINC, PORT, VCL, VMA1, VMA6, RHOG, SW, VO, WO, PORE, VXO, VW, AC1}
	Reduction attributes	{AC, GR, RI, RXO, SP}

**Table 15:** Performance of various well data.

Well	Classification model	RMSE	MAE	Accuracy (%)	Running time (s)
W1	RF	0.3326	0.1106	88.94	1.5167
	SVM	0.2681	0.0719	92.81	4.5861
	BSA-RF	0.3269	0.1069	89.31	1.8064
	DMSDL-BSA-RF	0.2806	0.0788	92.13	2.3728
	DMSDL-QBSA-RF	**0.2449**	**0.0600**	**94.00**	**1.4663**

W2	RF	0.4219	0.1780	82.20	3.1579
	SVM	0.2983	0.0890	91.10	4.2604
	BSA-RF	0.3963	0.1571	84.29	1.2979
	DMSDL-BSA-RF	0.2506	0.062827	93.72	1.6124
	DMSDL-QBSA-RF	**0.2399**	**0.0576**	**94.24**	**1.2287**

W3	RF	0.4028	0.1622	83.78	2.4971
	SVM	0.2507	0.0628	93.72	2.1027
	BSA-RF	0.3631	0.1318	86.81	1.3791
	DMSDL-BSA-RF	0.2341	0.0548	94.52	**0.3125**
	DMSDL-QBSA-RF	**0.0519**	**0.0027**	**99.73**	0.9513

## Data Availability

All data included in this study are available upon request by contact with the corresponding author.

## References

[1] Engelbrecht A. P. (2019). *Foundations of Computational Swarm Intelligence*.

[2] Kennedy J., Eberhart R. Particle swarm optimization.

[3] Karaboga D. (2005). An idea based on honey bee swarm for numerical optimization.

[4] Li X. (2003). *A new intelligent optimization method-artificial fish school algorithm*.

[5] Yang X. S. Firefly algorithms for multimodal optimization stochastic algorithms: foundations and applications.

[6] Sharafi Y., Khanesar M. A., Teshnehlab M. Discrete binary cat swarm optimization.

[7] Meng X. B., Gao X. Z., Lu L., Liu Y., Zhang H. Z. (2016). A new bio-inspired optimisation algorithm: bird swarm algorithm. *Journal of Experimental & Theoretical Artificial Intelligence*.

[8] Jatana N., Suri B. (2020). Particle swarm and genetic algorithm applied to mutation testing for test data generation: a comparative evaluation. *Journal of King Saud University-Computer and Information Sciences*.

[9] Chung H., Shin K. (2019). Genetic algorithm-optimized multi-channel convolutional neural network for stock market prediction. *Neural Computing and Applications*.

[10] Strumberger I., Tuba E., Zivkovic M., Bacanin N., Beko M., Tuba M. Designing convolutional neural network architecture by the firefly algorithm.

[11] Strumberger I., Bacanin N., Tuba M., Tuba E. (2019). Resource scheduling in cloud computing based on a hybridized whale optimization algorithm. *Applied Sciences*.

[12] Tuba M., Bacanin N. (2014). Improved seeker optimization algorithm hybridized with firefly algorithm for constrained optimization problems. *Neurocomputing*.

[13] Strumberger I., Minovic M., Tuba M., Bacanin N. (2019). Performance of elephant herding optimization and tree growth algorithm adapted for node localization in wireless sensor networks. *Sensors*.

[14] Yang X. S. (2014). Swarm intelligence based algorithms: a critical analysis. *Evolutionary Intelligence*.

[15] Bacanin N., Tuba M. (2012). Artificial bee colony (ABC) algorithm for constrained optimization improved with genetic operators. *Studies in Informatics and Control*.

[16] Liu J., Peng H., Wu Z., Chen J., Deng C. (2010). *Multi-Strategy Brainstorm Optimization Algorithm with Dynamic Parameters Adjustment*.

[17] Peng H., He Y., Deng C. (2019). Firefly algorithm with luciferase inhibition mechanism. *IEEE Access*.

[18] Peng H., Deng C., Wu Z. (2019). Best neighbor guided artificial bee colony algorithm for continuous optimization problems. *Soft Computing*.

[19] Xu C., Yang R. (2017). Parameter estimation for chaotic systems using improved bird awarm algorithm. *Modern Physics Letters B*.

[20] Yang J., Liu Y. Separation of signals with same frequency based on improved bird swarm algorithm.

[21] Wang X., Deng Y., Duan H. (2018). Edge-based target detection for unmanned aerial vehicles using competitive bird swarm algorithm. *Aerospace Science & Technology*.

[22] Breiman L. (2001). Random forests. *Machine Learning*.

[23] Ma L., Fan S. (2017). CURE-SMOTE algorithm and hybrid algorithm for feature selection and parameter optimization based on random forests. *BMC Bioinformatics*.

[24] Hou J., Li Q., Meng S. (2019). A differential privacy protection random forest. *IEEE Access*.

[25] Liu G. L., Han S. J., Zhao X. H. (2006). Optimisation algorithms for spatially constrained forest planning. *Ecological Modelling*.

[26] Gong D., Xu B., Zhang Y. A similarity-based cooperative co-evolutionary algorithm for dynamic interval multi-objective optimization problems.

[27] Rong M., Gong D., Pedrycz W. (2020). A multi-model prediction method for dynamic multi-objective evolutionary optimization. *IEEE Transactions on Evolutionary Computation*.

[28] Xu B., Zhang Y., Gong D., Guo Y., Rong M. (2018). Environment sensitivity-based cooperative co-evolutionary algorithms for dynamic multi-objective optimization. *IEEE-ACM Transactions on Computational Biology and Bioinformatics*.

[29] Guo Y., Yang H., Chen M., Cheng J., Gong D. (2019). Ensemble prediction-based dynamic robust multi-objective optimization methods. *Swarm and Evolutionary Computation*.

[30] Liang J. J., Suganthan P. N. Dynamic multi-swarm particle swarm optimizer with local search.

[31] Chen Y., Li L., Peng H. (2018). Dynamic multi-swarm differential learning particle swarm optimizer. *Swarm and Evolutionary Computation*.

[32] Storn R. Differential evolution design of an IIR-filter.

[33] Liu Z., Ji X., Yang Y. (2019). Hierarchical differential evolution algorithm combined with multi-cross operation. *Expert Systems with Applications*.

[34] Peng H., Guo Z., Deng C. (2018). Enhancing differential evolution with random neighbors based strategy. *Journal of Computational Science*.

[35] Sun J., Fang W., Wu X., Palade V., Xu W. (2012). Quantum-behaved particle swarm optimization: analysis of individual particle behavior and parameter selection. *Evolutionary Computation*.

[36] Chen B., Lei H., Shen H., Liu Y., Lu Y. (2019). A hybrid quantum-based PIO algorithm for global numerical optimization. *Science China–Information Sciences*.

[37] Yao X., Liu Y., Lin G. (1999). Evolutionary programming made faster. *IEEE Transactions on Evolutionary Computation*.

[38] Mirjalili S., Mirjalili S. M., Lewis A. (2014). Grey wolf optimizer. *Advances in Engineering Software*.

[39] Mirjalili S., Lewis A. (2016). The whale optimization algorithm. *Advances in Engineering Software*.

[40] Mirjalili S. (2016). SCA: a sine cosine algorithm for solving optimization problems. *Knowledge Based Systems*.

[41] Shahrzad S., Seyedali M., Andrew L. (2017). Grasshopper optimisation algorithm: theory and application. *Advances in Engineering Software*.

[42] Xue J., Shen B. (2020). A novel swarm intelligence optimization approach: sparrow search algorithm. *Systems Science & Control Engineering An Open Access Journal*.

[43] Bai J., Xia K., Lin Y., Wu P. (2017). Attribute reduction based on consistent covering rough set and its application. *Complexity*.

